# Airway epithelial cell identity and plasticity are constrained by Sox2 during lung homeostasis, tissue regeneration, and in human disease

**DOI:** 10.1038/s41536-023-00344-w

**Published:** 2024-01-05

**Authors:** Kazushige Shiraishi, Michael P. Morley, Dakota L. Jones, Gan Zhao, Aaron I. Weiner, Maria C. Basil, Edward Cantu, Laura T. Ferguson, Michele Oyster, Apoorva Babu, Yun Ying, Su Zhou, Shanru Li, Andrew E. Vaughan, Edward E. Morrisey

**Affiliations:** 1 Department of Medicine, Perelman School of Medicine, University of Pennsylvania, Philadelphia, PA 19104 USA; 2 Penn-CHOP Lung Biology Institute, Perelman School of Medicine, University of Pennsylvania, Philadelphia, PA 19104 USA; 3 Penn Cardiovascular Institute, University of Pennsylvania, Philadelphia, PA 19104 USA; 4 Department of Biomedical Sciences, School of Veterinary Medicine, University of Pennsylvania, Philadelphia, PA 19104 USA; 5 Institute for Regenerative Medicine, University of Pennsylvania, Philadelphia, PA 19104 USA; 6 Division of Cardiovascular Surgery, Department of Surgery, Perelman School of Medicine, University of Pennsylvania, Philadelphia, PA 19104 USA; 7 Department of Cell and Developmental Biology, Perelman School of Medicine, University of Pennsylvania, Philadelphia, PA 19104 USA

**Keywords:** Regeneration, Stem-cell differentiation

## Abstract

Maintenance of the cellular boundary between airway and alveolar compartments during homeostasis and after injury is essential to prohibit pathological plasticity which can reduce respiratory function. Lung injury and disease can induce either functional alveolar epithelial regeneration or dysplastic formation of keratinized epithelium which does not efficiently contribute to gas exchange. Here we show that Sox2 preserves airway cell identity and prevents fate changes into either functional alveolar tissue or pathological keratinization following lung injury. Loss of Sox2 in airway epithelium leads to a loss of airway epithelial identity with a commensurate gain in alveolar and basal cell identity, in part due to activation of Wnt signaling in secretory cells and increased Trp63 expression in intrapulmonary basal-like progenitors. In idiopathic pulmonary fibrosis, loss of SOX2 expression correlates with increased WNT signaling activity in dysplastic keratinized epithelium. SOX2-deficient dysplastic epithelial cells are also observed in COVID-19 damaged lungs. Thus, Sox2 provides a molecular barrier that suppresses airway epithelial plasticity to prevent acquisition of alveolar or basal cell identity after injury and help guide proper epithelial fate and regeneration.

## Introduction

The primary function of the lung is to facilitate gas exchange through interactions of the complex epithelial surface with the pulmonary vasculature. While the lung is generally a quiescent organ during homeostasis, it possesses the ability to regenerate in response to severe tissue damage, such as that caused by influenza or COVID-19 infection^1^. During lung alveolar regeneration due to severe injury, multiple cell lineages contribute to at least two major modes of regeneration: 1) functional regeneration, in which alveolar type 2 (AT2) cells proliferate and differentiate into alveolar type 1 (AT1) epithelial cells, and 2) dysplastic regeneration, which leads to the formation of bronchiolar-like clusters of keratinized cells often called keratin “pods”^2–4^. Previous work using various lineage mapping techniques has shown that the airway-derived functional alveolar regeneration is promoted by distal secretory cells^5–7^ and that keratinized pods are derived from distal airway epithelial cells expressing Sox2 and/or Trp63 in mice^2–4,8–10^. These keratin pods do not appear to contribute to gas exchange and persist indefinitely in the lungs after the initial injury, resulting in epithelial “scar tissue” that is detrimental in the long-term^4,8,9^. Recent studies have also demonstrated that distal airway epithelial cells can generate alveolar epithelium after severe lung injury^2,5^. However, the molecular determinants of distal airway cell fate maintenance during lung regeneration and how these cells alter their fate decisions to generate either alveolar or keratinized epithelium are not fully understood.

Mouse airways are structured differently than human airways with Trp63^+^ basal cells found in the trachea and main stem bronchi but only rarely in the intrapulmonary airways. Mouse airways also end abruptly upon entering the alveolar compartment at a region called the bronchoalveolar junction, where a few rare epithelial cells called bronchioalveolar stem cells (BASCs) are located^6,7,11^. BASCs are identified by their co-expression of certain secretory cell and AT2 cell markers such as Scgb1a1 and Sftpc, respectively, and can generate both airway and alveolar epithelial lineages after lung injury. In the human lung, the distal airways ramify to interdigitate with alveolar tissue in a structure called respiratory bronchioles (RBs)^12,13^. Human RBs lack extensive investment by basal cells. Rather, they are populated by a specialized respiratory airway secretory cell (RASC), which can generate both airway and alveolar epithelial cell lineages^14–16^. Thus, in both mouse and human airways, specialized airway epithelial cells can generate alveolar epithelium depending on the injury.

Recent studies have shown that activation of Notch and hypoxic signaling enhances dysplastic keratinized regeneration in the lung, while activation of Wnt signaling promotes functional alveolar epithelial regeneration by airway cells^4,17^. However, the transcriptional mechanisms that regulate airway versus alveolar epithelial fate are less clear. Moreover, how airway cells maintain their fate and distinctive phenotype at homeostasis is poorly understood. Sox2 is expressed at high levels in airway epithelial cells of the mouse and human respiratory system but is not expressed in alveolar epithelium of the mouse^18,19^. While previous work has shown that Sox2 is required for normal airway epithelial development and regeneration of luminal tracheal epithelium^19–21^, whether Sox2 plays a role in balancing airway versus alveolar epithelial cell fate during lung regeneration is unknown.

In this study, we reveal that Sox2 serves as a molecular barrier to both alveolar and basal cell fates in the lung. Using a multi-modal analysis, we show that Sox2 plays a crucial role in maintaining airway cell identity and preventing fate changes during lung regeneration. Loss of Sox2 expression leads to a loss of airway cell identity at homeostasis and enhances airway cell fate reprogramming into both alveolar epithelial cells and keratinized Trp63^+^ cells following influenza infection. Single cell RNA-seq (scRNA-seq) analysis shows that Sox2-deficient basal-like progenitor cells exhibit elevated expression of the Trp63 dependent basal cell program, while Sox2-deficient airway secretory cells exhibit elevated expression of the AT2 cell program. Loss of Sox2 augments formation of dysplastic keratinized epithelium after injury in the distal lung and enhances alveolar differentiation cooperatively with Wnt signaling. SOX2 deficient dysplastic epithelium is found in lungs from both idiopathic pulmonary fibrosis and COVID-19 patients, suggesting conservation of this molecular barrier function across species. Together, these studies indicate that Sox2 functions as a molecular barrier that inhibits airway epithelial plasticity by preventing the acquisition of alveolar or basal cell identity after injury, which is critical for directing proper epithelial fate and facilitating regeneration.

## Results

### Sox2 maintains airway cell identity

**Fig. 1:**
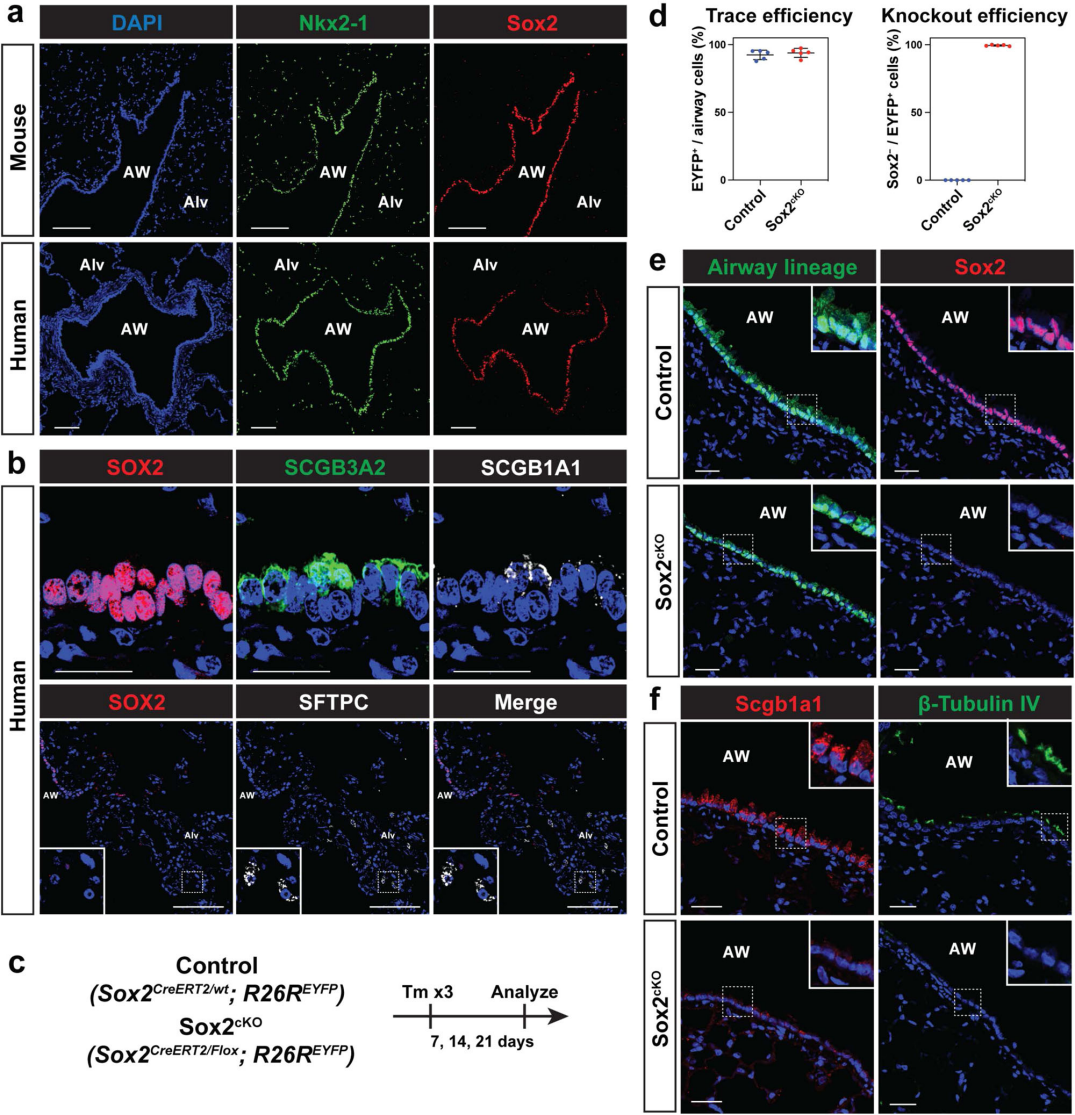
Deletion of Sox2 disrupts the maintenance of airway epithelial cell identity. **a** Positive Sox2 expression distinguishes airway epithelial cells from alveolar epithelial cells. Nkx2-1^+^ Alveolar cells do not express Sox2. AW denotes airway, and Alv indicates alveoli. **b** SCGB3A2^+^ SCGB1A1^high^ and SCGB3A2^+^ SCGB1A1^low^ human secretory cells express SOX2 (*n* = 4 healthy human samples) but SFTPC^+^ AT2 cells do not. **c**–**e** Efficient lineage tracing and Sox2 deletion using *Sox2*^*CreERT2/Flox*^*; R26R*^*EYFP*^ mice. *Sox2*^*CreERT2/wt*^*; R26R*^*EYFP*^ (control) and *Sox2*^*CreERT2/Flox*^*; R26R*^*EYFP*^ (Sox2^cKO^) mice were given tamoxifen and analyzed 7, 14, and 21 days later (**c**). Quantification for lineage-trace efficiency and Sox2 deletion in control and Sox2^cKO^ mice (**d**). Representative images showing deletion of Sox2 in Sox2^cKO^ mice (**e**). Each dot represents an individual mouse, and error bars indicate mean with s.d. *N* = 5 mice for control and Sox2^cKO^. **f** Secretory cell marker Scgb1a1 and ciliated cell marker β-Tubulin IV are downregulated in Sox2^cKO^ mice. Images obtained from mice 21 days after tamoxifen administration. Scale bars: **a**, **b** (bottom) 100 μm; **b** (top), **e**, **f** 25 μm.

Sox2 expression is observed in airway but not alveolar epithelium in the mouse and human adult lung (Fig. 1a). In the adult human terminal and respiratory bronchioles, SCGB3A2^+^/SCGB1A1^high^ and SCGB3A2^+^/SCGB1A1^low^ populations also expresses SOX2, while SFTPC^+^ human AT2 cells do not express SOX2 (Fig. 1b)^14–16^. To determine the role for Sox2 in airway cell homeostasis, we generated *Sox2*^*CreERT2/Flox*^*; R26R*^*EYFP*^ (hereafter called Sox2^cKO^) mice to inactivate Sox2 in adult airway epithelial cells and analyzed these mice 7, 14, and 21 days after tamoxifen administration (Fig. 1c and Supplementary Fig. 1a). Efficient lineage-tracing and Sox2 deletion were confirmed by immunohistochemistry (IHC) (Fig. 1d, e). IHC for the secretory cell marker Scgb1a1 and multi-ciliated cell marker β-Tubulin IV revealed a dramatic loss of their expression upon loss of Sox2 expression as early as 7 days after tamoxifen administration, suggesting the loss of airway luminal epithelial cell identity (Fig. 1f and Supplementary Fig. 1b). Sox2-deficient airway cells do not express the basal cell marker Krt5, suggesting that Sox2 deletion does not cause mucocilliary cells to be reprogrammed into basal stem cells, but rather that these cells lose their original identity (Supplementary Fig. 1b). Cell density and cell height were decreased in Sox2^cKO^ mice, which was also observed in a previous report using Scgb1a1-Cre mice, suggesting morphological changes in airway cells (Supplementary Fig. 1c, d)^22^. These findings suggest that Sox2 maintains secretory and multi-ciliated cell identity in mouse intrapulmonary airways.

### Sox2 transcriptionally restricts both alveolar and basal cell fates in mouse intrapulmonary airways

**Fig. 2:**
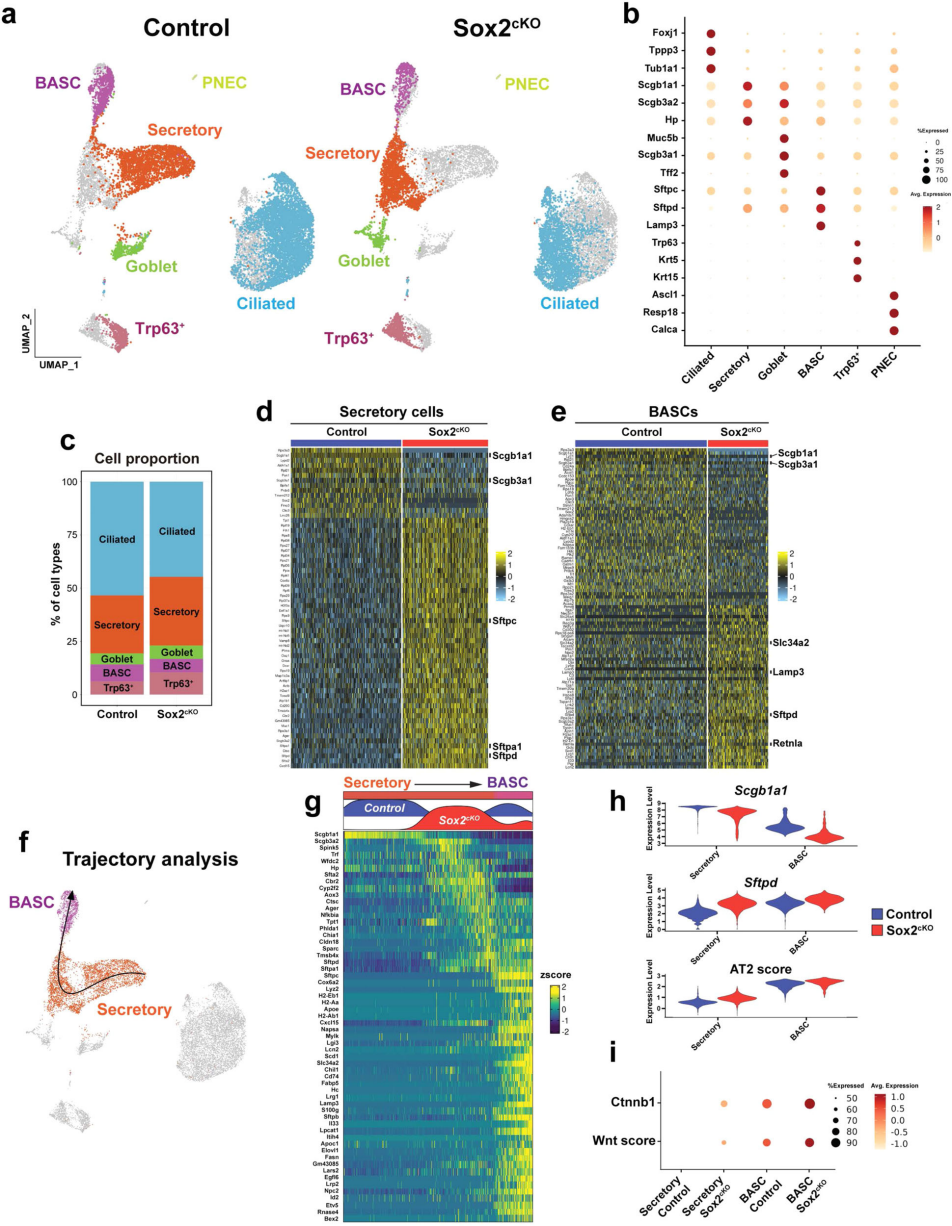
scRNA-seq analysis reveals acquisition of alveolar signature in Sox2-deficient airway cells. **a** Airway cells from control and Sox2^cKO^ mice form distinct cell populations on the UMAP plot. Individual sample was plotted in the joint UMAP. **b** Dotplot showing canonical cell marker gene expressions in each cell population. Marker genes were extracted from CellCards. Both control and Sox2-deficient cells were used for plotting. **c** Cell proportions in control and Sox2^cKO^ mice. Heatmaps showing the top 50 differentially expressed genes for secretory cells (**d**) and BASCs (**e**). **f** Trajectory analysis shows lineage relationships between Sox2^cKO^ secretory cells and BASCs. **g** Heatmap showing expression of genes defining trajectory. **h** Scgb1a1 was downregulated and Sftpd was upregulated in secretory cells and BASCs of the Sox2^cKO^ mice. AT2 score, generated using AT2 cell marker genes, was higher in secretory cells and BASCs. **i** Ctnnb1 was upregulated in secretory cells and BASCs of the Sox2^cKO^ mice. Wnt activation score, generated using Wnt downstream genes, was higher in Sox2^cKO^ mice. Wnt downstream genes were obtained from “the Wnt homepage”.

To assess the transcriptional changes that underlie these changes in cell fate, we performed single cell RNA-sequencing (scRNA-seq) on lineage-traced cells from the lung lobes of control and Sox2^cKO^ mice (Fig. 2a and Supplementary Fig. 2a). In both control and Sox2^cKO^ mice, we detected secretory, ciliated, goblet, Trp63^+^ intrapulmonary basal-like progenitor, pulmonary neuroendocrine, and bronchioalveolar stem cells (BASCs) (Fig. 2a, b). Control BASCs were defined in this dataset by their co-expression of secretory and AT2 cell markers along with Sox2, whereas Sox2-deficient BASCs were defined based on their expression of secretory and AT2 cell markers and co-clustering with control BASCs (Fig. 2b and Supplementary Fig. 2b). Control and Sox2^cKO^ secretory, goblet, and basal-like progenitor lineages occupied different transcriptional space in the UMAP presentation, revealing distinct transcriptional signatures (Fig. 2a). The percentage of each population among all cell types was not significantly altered in Sox2^cKO^ mice (Fig. 2c).

Using trajectory inference analysis to reveal pseudotemporal relationship between control and Sox2^cKO^ secretory cells with BASC cells, we found that the transcriptome of secretory cells in Sox2^cKO^ mice was shifted towards a BASC-like signature (Fig. 2d, f, g). This included decreased expression of canonical secretory cell genes such as Scgb1a1 and increased expression of AT2 cell genes such as Lamp3 and Sftpd (Fig. 2d, g, h, and Supplementary Data 1). We developed an AT2 signature gene list and scored secretory and BASC cells in control and Sox2^cKO^ mice, which revealed an increase in the AT2 transcriptional signature in both of these lineages in Sox2^cKO^ mice (Fig. 2e, h, and Supplementary Table 1). Wnt/β-catenin signaling plays critical roles in alveolar epithelial cell fate decisions including promoting the AT2 cell fate during lung regeneration^23,24^. We observed upregulation of Ctnnb1 and Wnt pathway downstream genes, as defined by genes listed in the target genes section of the Wnt homepage (https://web.stanford.edu/group/nusselab/cgi-bin/wnt/), in secretory and BASC lineages, which could explain the acquisition of alveolar signatures (Fig. 2i and Supplementary Table 1). Interestingly, goblet cells also showed a decrease in Scgb1a1 and an increase in AT2 cell genes, such as Sftpd and Lyz2 (Supplementary Fig. 3a and Supplementary Data 1). The expression of Sox9, a transcription factor associated with alveolar epithelial fate choice during early lung development^25^, was not altered in Sox2^cKO^ airway cells (Supplementary Fig. 3b, c). The expression of Sox21, a transcription factor recently reported to have roles during airway development and regeneration^26^, was not increased in Trp63^+^ intrapulmonary basal-like progenitors (Supplementary Fig. 3d).

**Fig. 3:**
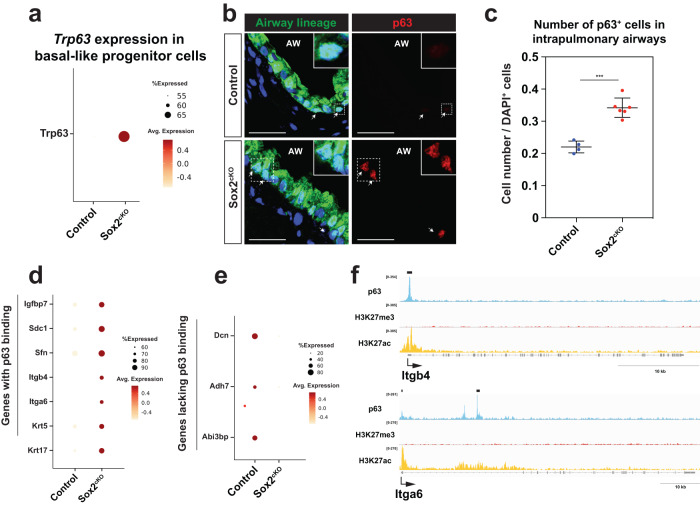
Sox2 deletion upregulates basal signature in basal-like progenitors. **a** Trp63 expression is upregulated in Trp63^+^ intrapulmonary basal-like progenitor cells of Sox2^cKO^. **b**, **c** p63 is upregulated in basal-like progenitors of Sox2^cKO^ mice (**b**). The number of Intrapulmonary basal-like progenitors increase in Sox2^cKO^ mice (*n* = 6 mice) compared to control mice (*n* = 4 mice). Quantification was performed in intrapulmonary airways within 1-2 bifurcations of the right or left intrapulmonary bronchus (**c**). Scale bars: 25 μm. **d**–**f** Basal cell genes reported to have p63 ChIP-seq peaks were upregulated (**d**) and basal cell genes without p63 ChIP-seq peaks were downregulated (**e**) in p63^+^ intrapulmonary basal-like progenitor cells from Sox2^cKO^ mice. ChIP tracks showing p63 peaks for Itgb4 and Itga6 (**f**). ChIP-seq data were obtained from Weiner et al. ^9^. ***P < 0.001 by two-tailed t-test. Each dot represents an individual mouse, and error bars indicate mean with s.d.

Intrapulmonary basal-like progenitor lineage is the only cell lineage in the distal lung that expresses the basal cell master transcription factor Trp63. As the basal-like progenitor cell lineage also exhibited transcriptional changes upon loss of Sox2 expression, we analyzed Trp63 expression which revealed an increase in Trp63 expression in intrapulmonary basal-like progenitor cells of Sox2^cKO^ mice (Fig. 3a). Moreover, there was an increase in the number of Trp63^+^ basal-like progenitor cells in the intrapulmonary airways of Sox2^cKO^ mice (Fig. 3b, c). Expression of many basal cell marker genes that are directly regulated by Trp63 as noted by ChIP-seq analysis, such as Itgb4 and Itga6, are also increased in basal-like progenitor cells in the intrapulmonary airways of Sox2^cKO^ mice (Fig. 3d, f)^9^. Interestingly, some basal cell genes not known to be regulated by Trp63 decreased in Sox2-deficient basal-like progenitor cells (Fig. 3e). These data suggest that airway cell identity is maintained at homeostasis by Sox2 and upon its loss, secretory cells acquire a more BASC/AT2 signature while basal-like progenitor cells increase Trp63 expression and the number of basal-like progenitor cells in the proximal intrapulmonary airways.

### Sox2 mediates alveolar regeneration following severe lung injury

**Fig. 4:**
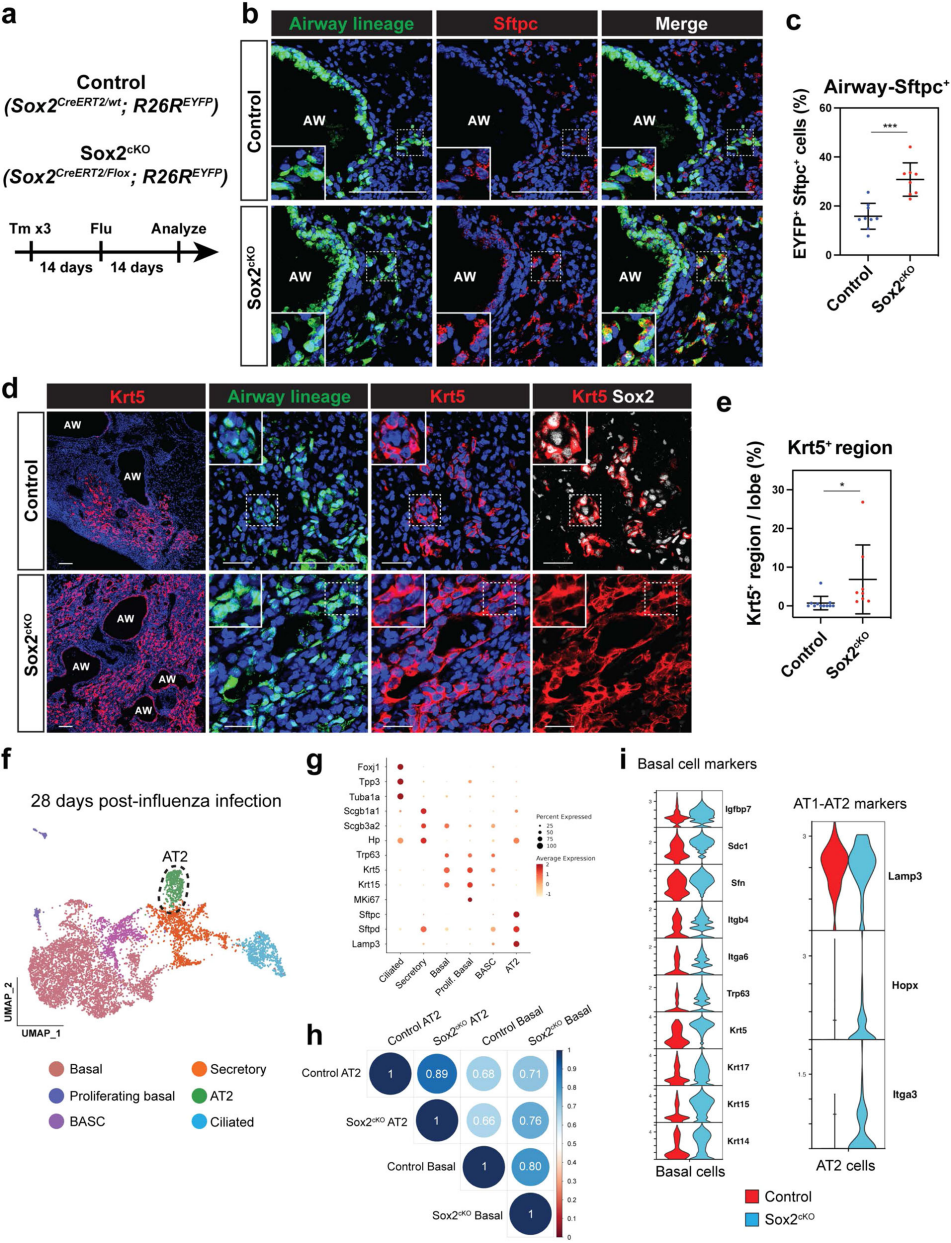
Sox2 deletion promotes airway cell contribution to alveolar regeneration. **a** Influenza virus was intranasally administered to control and Sox2^cKO^ mice and the mice were analyzed 14 days after the injury. **b**, **c** Sox2 deletion promotes airway cell transition to Sftpc^+^ AT2 cells after influenza lung injury. EYFP^+^ lineage-traced airway cells transition to Sftpc^+^ AT2 cells in control (**b** top), but more so in the Sox2^cKO^ mice (**b** bottom) after injury. Quantification for the percentage of lineage-traced EYFP^+^ Sftpc^+^ cells after influenza lung injury (*n* = 9 control and *n* = 8 Sox2^cKO^ mice from 3 independent experiments) (**c**). **d**, **e** Sox2 deletion promotes airway cell transition to dysplastic keratinized pods after influenza lung injury. EYFP^+^ lineage-traced airway cells transition to Krt5^+^ dysplastic keratinized cells in control (**d** top), but more so in the Sox2^cKO^ mice (**d** bottom) after injury. Quantification for the area size of the Krt5^+^ region after influenza lung injury (*n* = 11 control and *n* = 8 Sox2^cKO^ mice from 3 independent experiments) (**e**). **f** Joint UMAP showing lineage traced populations of EYFP^+^ cells from control and Sox2^cKO^ mice 28 days after influenza infection. **g** Dotplot showing canonical cell marker gene expressions in each cell population. **h** Correlation analysis shows that AT2 and basal cells derived from either control or Sox2^cKO^ airway cells were similar. **i** Expression of basal cell genes and alveolar epithelial marker genes in control and Sox2^cKO^ mice 28 days after influenza infection. ***P < 0.001 and *P < 0.05 by two-tailed t-test. N.S. not significant. Each dot represents an individual mouse, and error bars indicate mean with s.d. Scale bars: **b**, **d** 100 μm.

The scRNA-seq analysis suggested that loss of Sox2 shifts the fate of airway epithelium towards an alveolar fate at homeostasis. To determine what affect this would have after acute injury, Sox2^cKO^ and control mice were subjected to influenza lung injury and analyzed 14 days after the injury when active epithelial regeneration is occurring (Fig. 4a)^4^. We found that the percentage of Sox2^+^ lineage-traced AT2 cells increased significantly in Sox2^cKO^ mice, indicating that Sox2 restricts alveolar fate after injury (Fig. 4b, c). We also found lineage-traced AT1 cells, suggesting that airway-derived AT2 cells can differentiate into AT1 cells (Supplementary Fig. 4a). Moreover, loss of Sox2 increased the formation of Krt5^+^ epithelium likely due to increased expression and numbers of Trp63^+^ basal-like progenitor cells in the intrapulmonary airways, which are precursors to dysplastic regeneration in the lung (Fig. 4d, e)^9^. Krt5^+^ p63^+^ epithelium does not express AT2 cell marker Lamp3, and airway-derived AT2 cells do not express Krt5 or p63, confirming that Krt5^+^ epithelium and airway-derived AT2 cells are distinct cell types (Supplementary Fig. 4b, c). No differences in body weight changes or oxygen saturation were observed between control and Sox2^cKO^ mice, suggesting that Sox2 pan-airway knockout alone does not alter the overall regenerative benefits in the lungs (Supplementary Fig. 4d, e).

We next assessed any changes in AT2 and basal cells from control and Sox2^cKO^ mice upon influenza injury using scRNA-seq analysis. scRNA-seq analysis at 28 days after influenza infection shows that Sox2 lineage-traced cells can generate bona fide AT2 cells, as indicated by expression of canonical AT2 markers such as Sftpc and Lamp3, but with very low levels of secretory cell markers such as Scgb1a1 (Fig. 4f, g, and Supplementary Fig. 4f). Using Spearman correlation analysis, AT2 and basal cells derived from either control or Sox2^cKO^ airway cells were similar (Fig. 4h). However, there was a marked increase in basal cell marker genes in Sox2-deficient basal cells (Fig. 4i and Supplementary Data 2). Moreover, there was a marked increase in some AT1 markers in Sox2 deficient airway-derived AT2 cells, suggesting an accelerated rate of alveolar epithelial differentiation after injury (Fig. 4i and Supplementary Data 2). Secretory cells from Sox2^cKO^ mice showed higher expression of transition state AT2 cell markers^27–29^, further suggesting the accelerated differentiation (Supplementary Fig. 4g). These results reveal that Sox2 restricts AT2 and basal cell fate during both homeostasis and acute injury, leading to enhanced airway contributions towards alveolar regeneration as well as dysplastic Krt5^+^ epithelial formation.

### Sox2 deletion leads to airway-derived alveolar regeneration through active Wnt signaling

Wnt/β-catenin signaling is known to play key roles in lung development from lung endoderm specification through balancing AT2 cell self-renewal and AT2-AT1 cell differentiation^23,30^. Moreover, activation of Wnt signaling has been shown to inhibit the formation of keratinized dysplastic epithelium after acute lung injury^17^. Previous work has shown that many SOX transcription factors physically interact with β-catenin including Sox2, which can downregulate Wnt pathway-responsive genes by blocking nuclear localization of β-catenin and preventing it from binding to TCF/LEF factors^31,32^.

To evaluate the role of Wnt signaling during airway-to-alveolar cell transition in airway epithelium, airway organoids were generated from FACS sorted control and Sox2^cKO^ lung airway epithelium. After two weeks in culture, airway cells generated organoids composed of Krt5^+^ basal cells and Tubulin IV^+^ ciliated cells, and alveolar organoids composed of Sftpc^+^ AT2 and Hopx^+^ AT1 cells (Supplementary Fig. 5a, b)^33,34^. To determine whether Wnt signaling was necessary for this alveolar differentiation in airway epithelium, organoids were cultured in the presence or absence of the tankyrase inhibitor XAV939. XAV939-treated organoids generated fewer alveolar organoids in both control and Sox2^cKO^ (Supplementary Fig. 5c). This analysis showed that Wnt signaling is critical for Sox2^+^ airway cell differentiation into alveolar epithelial cells.

**Fig. 5:**
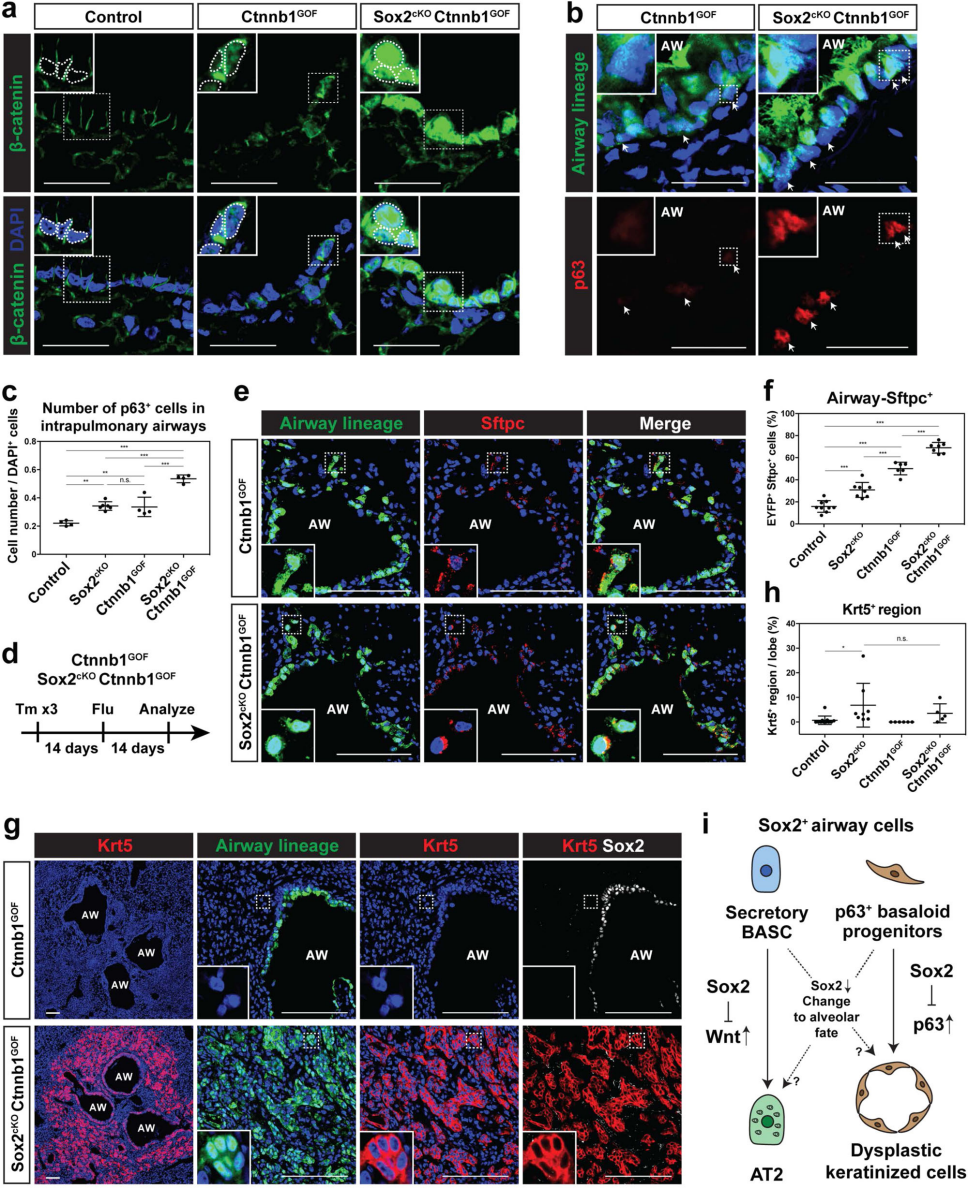
Sox2 deletion leads to airway cell contribution to alveolar regeneration in coordination with Wnt signaling. **a** Expression of nuclear β-catenin is more prominent in airways of Sox2^cKO^/Ctnnb1^GOF^ mice than Ctnnb1^GOF^ or control wild-type mice. **b** Intrapulmonary basal-like progenitors from Sox2^cKO^/Ctnnb1^GOF^ mice express more p63 than Ctnnb1^GOF^ mice. **c** The number of intrapulmonary basal-like progenitors increase in Sox2^cKO^/Ctnnb1^GOF^ mice (*n* = 4 mice) compared to Ctnnb1^GOF^ mice (*n* = 4 mice). Quantification was performed in intrapulmonary airways within 1-2 bifurcations of the right or left intrapulmonary bronchus. The results from Fig. 3c are reused for comparison. **d** Influenza virus was intranasally administered to control Ctnnb1^GOF^ and Sox2^cKO^/Ctnnb1^GOF^ mice and the mice were analyzed 14 days after the injury. **e**, **f** Sox2 deletion promotes airway cell transition to Sftpc^+^ AT2 cells after influenza lung injury in Ctnnb1^GOF^ mice. EYFP^+^ lineage-traced airway cells transition to Sftpc^+^ AT2 cells in control Ctnnb1^GOF^ (**e** top), but more so in the Sox2^cKO^/Ctnnb1^GOF^ mice (**e** bottom) after injury. Quantification for the percentage of lineage-traced EYFP^+^ Sftpc^+^ cells after influenza lung injury (*n* = 6 control and *n* = 7 Sox2^cKO^/Ctnnb1^GOF^ mice from 2 independent experiments) (**f**). The results from Fig. 4c, which are from different experiments, are reused for comparison. **g**, **h** Sox2 deletion promotes airway cell transition to dysplastic keratinized pods after influenza lung injury in Ctnnb1^GOF^ mice. Krt5^+^ pods were not formed in control (**g** top), but the pods were observed in the Sox2^cKO^/Ctnnb1^GOF^ mice (**g** bottom) after injury. Quantification for the area size of the Krt5^+^ region after influenza lung injury (*n* = 6 control and *n* = 5 Sox2^cKO^/Ctnnb1^GOF^ mice from 2 independent experiments) (**h**). The results from Fig. 4e, which are from different experiments, are reused for comparison. **i** Model describing the loss of Sox2 and airway fate decisions after lung injury. ***P < 0.001 and **P < 0.01 by one-way ANOVA. Each dot represents an individual mouse, and error bars indicate mean with s.d. Scale bars: **e**, **g** 100 μm; **a**, **b** 25 μm.

To determine whether Wnt signaling enhanced the ability of Sox2-deficient airway epithelium to differentiate into alveolar epithelium in vivo, we generated *Sox2*^*CreERT2/wt*^*; Ctnnb1*^*flox(ex3)/wt*^*; R26R*^*EYFP*^ (Ctnnb1^GOF^) mice to activate Wnt/β-catenin signaling in airway cells and *Sox2*^*CreERT2/Flox*^*; Ctnnb1*^*flox(ex3)/wt*^*; R26R*^*EYFP*^ (hereafter Sox2^cKO^/Ctnnb1^GOF^) mice to simultaneously inactivate Sox2 and activate Wnt/β-catenin signaling in airway cells. While increased β-catenin expression was observed in Ctnnb1^GOF^ airway epithelium, a dramatic increase in nuclear localized β-catenin was observed in Sox2^cKO^/Ctnnb1^GOF^ animals (Fig. 5a). Sox2^cKO^/Ctnnb1^GOF^ airway cells also express more Sftpc than Ctnnb1^GOF^ airway cells even at homeostasis (Supplementary Fig. 5d, e). Expression of the basal cell marker Trp63 is also increased in intrapulmonary basal-like progenitor cells of Sox2^cKO^/Ctnnb1^GOF^ as compared to Ctnnb1^GOF^ mice (Fig. 5b). The number of basal-like progenitor cells was increased in Sox2^cKO^/Ctnnb1^GOF^ compared to Ctnnb1^GOF^ (Fig. 5c). The Ctnnb1^GOF^ mutation alone increased the number of basal-like progenitors, suggesting that Ctnnb1^GOF^ and Sox2^cKO^ have additive or synergistic effects on the cells at homeostasis. Sox2^cKO^/Ctnnb1^GOF^ and Ctnnb1^GOF^ were subjected to influenza lung injury and analyzed 14 days after the injury (Fig. 5d). These data revealed that the percentage of Sox2^+^ lineage-traced Sftpc^+^ AT2 cells was higher in Sox2^cKO^/Ctnnb1^GOF^ mice compared to Ctnnb1^GOF^ mice (Fig. 5e, f). The percentage of Sox2^+^ lineage-traced Sftpc^+^ AT2 cells increased in Ctnnb1^GOF^, suggesting that Ctnnb1^GOF^ and Sox2^cKO^ have additive or synergistic effects. Importantly, formation of Krt5^+^ keratinized epithelium was observed in Sox2^cKO^/Ctnnb1^GOF^ but not in Ctnnb1^GOF^ mice (Fig. 5g, h), the latter in agreement with earlier studies^17^. Sox2 deletion and nuclear β-catenin were confirmed by IHC (Supplementary Fig. 5f, g). The formation of Krt5^+^ dysplastic epithelium in Sox2^cKO^/Ctnnb1^GOF^ mice and not Ctnnb1^GOF^ mice is likely due to the increased expression and presence of Trp63^+^ basal-like progenitor cells, as a previous study showed that Trp63 is essential for cell migration and formation of alveolar Krt5^+^ dysplastic epithelium (Fig. 5b, c)^9^. These results demonstrate that Sox2-Wnt interactions restrict airway epithelial fate at both poles of the proximal-distal airway axis in the mouse lung (Fig. 5i).

### Dysplastic regeneration in human lungs involves SOX2 deficient basaloid cells in pulmonary fibrosis and COVID-19

**Fig. 6:**
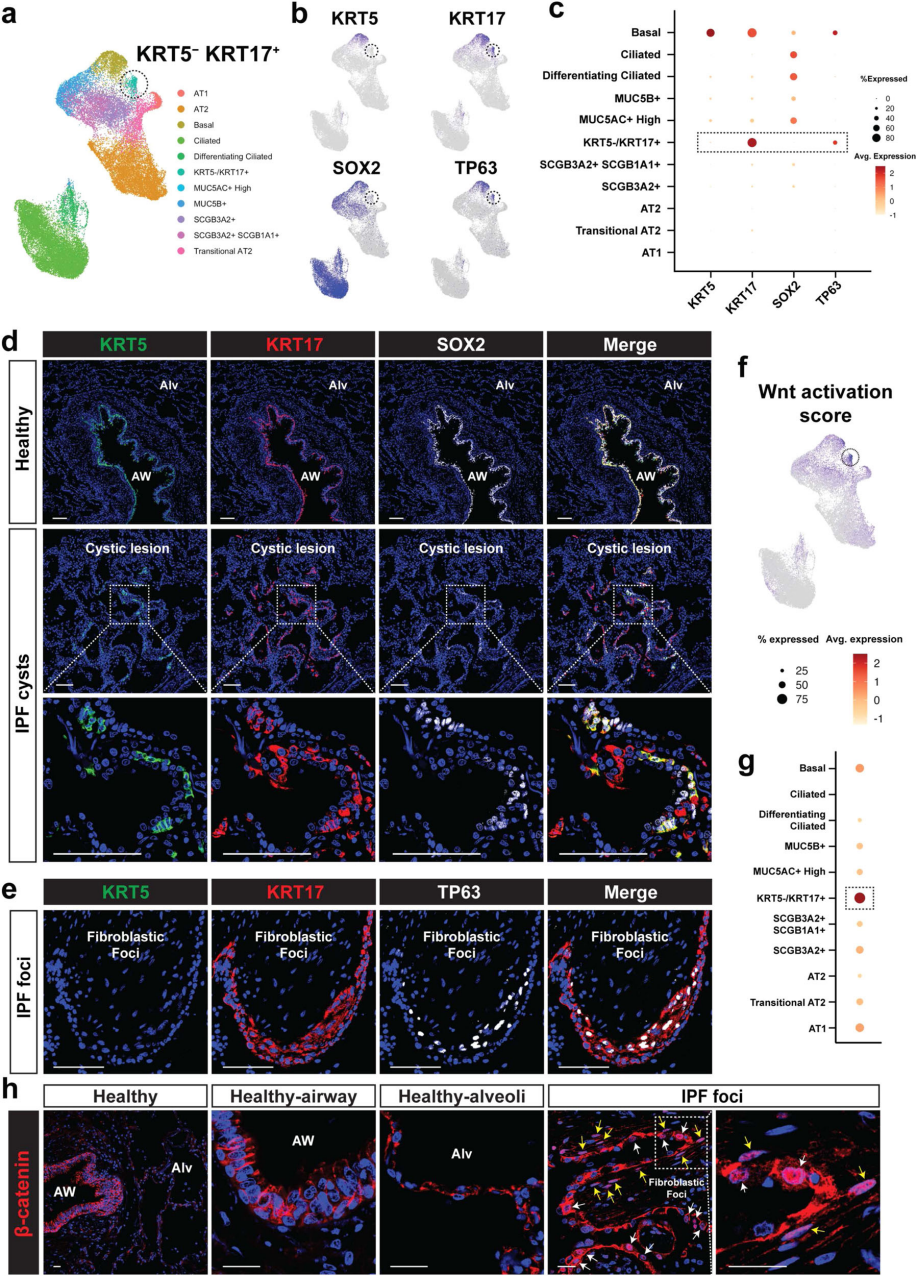
Identification of SOX2 deficient, WNT active, KRT5^–^/KRT17^+^ dysplastic epithelial cells in IPF. **a** UMAP plot showing epithelial cell clusters in patients with IPF. scRNA-seq Data obtained from Habermann et al.^35^. **b**, **c** KRT5^–^/KRT17^+^ dysplastic cells are TP63^+^ and SOX2^–^. UMAP plot (**b**) and Dotplot (**c**). **d** Immunohistochemistry analysis of human IPF showing negative SOX2 in KRT5^–^/KRT17^+^ dysplastic cells (*n* = 3 samples). **e** Immunohistochemistry analysis of human IPF showing positive TP63 in KRT5^–^/KRT17^+^ dysplastic cells (*n* = 2 samples). **f**, **g** Wnt activation score is higher in KRT5^–^/KRT17^+^ cells. Wnt downstream genes obtained from “the Wnt homepage” were used for scoring. UMAP plot (**f**) and Dotplot (**g**) are shown. **h** Immunohistochemistry analysis of human IPF showing nuclear β-catenin signal in the epithelial cells and spindle-shaped fibroblasts of fibroblastic foci. Nuclear β-catenin signal was not prominent in healthy human samples (*n* = 2 samples). White arrows denote epithelial cells and yellow arrows denote fibroblasts (*n* = 4 IPF samples). Scale bars: **d**, **e** 100 μm; **h** 25 μm.

**Fig. 7:**
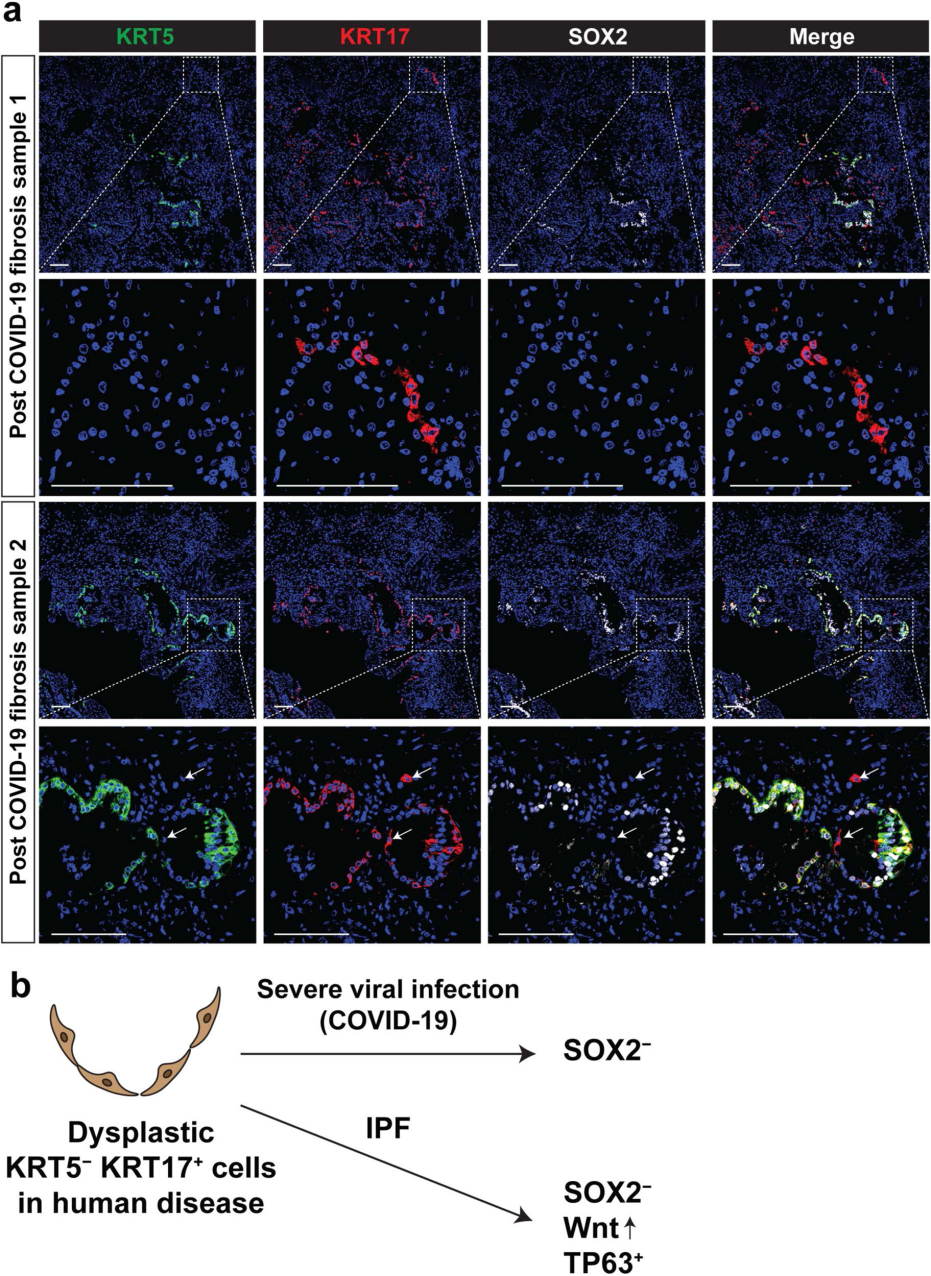
KRT17^+^ dysplastic epithelial cells in post-COVID-19 pulmonary fibrosis patients are SOX2 deficient. **a** Immunohistochemistry analysis of human post-COVID19 pulmonary fibrosis showing negative SOX2 in KRT5^–^/KRT17^+^ dysplastic cells (*n* = 3 samples). **b** Model describing negative expression of SOX2 in dysplastic KRT5^−^/KRT17^+^ cells in human pulmonary fibrosis. Scale bars: 100 μm.

The role of SOX2 and other SOX family members in human lung disease is largely unknown. Therefore, we analyzed scRNA-seq data from patients with pulmonary fibrosis, a disease known to involve dysplastic remodeling of the lung, including formation of keratinized epithelium in the distal parenchyma similar to what is observed after viral injury in mice (Supplementary Fig. 6a)^35^. IHC of samples from IPF patients revealed the presence of KRT5^+^/KRT17^+^/SOX2^+^ basal lineage cells and previously reported KRT5^−^/KRT17^+^ dysplastic epithelial cells (Fig. 6a–d and Supplementary Table 2)^9,35,36^. Interestingly, these KRT5^−^/KRT17^+^ cells in cystic regions express SOX2 at a lower level than secretory or multi-ciliated lineages and are present in lungs from IPF patients but not normal donors (Fig. 6b–d and Supplementary Fig. 6a, b). These KRT5^−^/KRT17^+^ cells also express TP63 (Fig. 6b, c, e). We next generated a gene expression score using known transcriptional targets of Wnt signaling to assess where active Wnt signaling occurs in the epithelium of IPF lungs. This analysis in combination with β-catenin IHC showed that the Wnt pathway is activated in the KRT5^−^/KRT17^+^ cell population to a far greater extent than in other epithelial lineages (Fig. 6f–h, Supplementary Fig. 7a, b, and Supplementary Table 1). Expression of several Wnt signaling components were also highly enriched in these cells including DVL3, TCF7L2 and CTNNB1 (Supplementary Fig. 7c). To determine whether KRT5^−^/KRT17^+^ cells could be found after an acute viral injury in the human lung, samples from patients who had severe COVID-19 disease were examined by IHC. These data revealed the presence of KRT5^−^/KRT17^+^ cells in COVID-19 patient lungs at time of transplant (Fig. 7a). These KRT5^−^/KRT17^+^ cells from COVID-19 patients were found to express SOX2 at a lower level than other cell types. The SOX2^−^/TP63^+^ Wnt-activated transcriptional signature of KRT5^−^/KRT17^+^ basaloid cells in IPF and SOX2^−^ signature of KRT5^−^/KRT17^+^ basaloid cells in post-COVID-19 fibrosis suggest that loss of SOX2 expression may explain the formation of these distinct dysplastic lesions in lung disease (Fig. 7b). Mouse Krt5^+^ pods also express Krt17, but we did not detect Krt5^−^ Krt17^+^ cells in mice, suggesting that there is a species-specific nature of keratin protein expression that may or may not be related to pathology (Supplementary Fig. 7d). Nevertheless, these results suggest that loss of SOX2 may contribute to Wnt activation and TP63 expression in KRT5^−^/KRT17^+^ dysplastic cells of IPF and pathological remodeling in human lung disease.

## Discussion

Our study highlights the crucial role of the transcription factor Sox2 in acting as a molecular barrier at apposing poles of the airway to restrict alveolar and basal epithelial cell fates. Deletion of Sox2 during homeostasis leads to down-regulation of secretory and multi-ciliated cell transcriptional programs with a commensurate up-regulation of alveolar and basal cell programs. After acute viral lung injury, loss of Sox2 enhances airway-to-alveolar epithelial fate transition and formation of Krt5^+^ dysplastic regeneration. The airway-to-alveolar epithelial fate transition is mediated by Wnt signaling, which is enhanced in secretory and BASC cells by the loss of Sox2. Wnt activation prior to injury inhibits dysplastic regeneration, however when combined with Sox2 deletion, enhances dysplastic regeneration mediated by Trp63^+^ cells. In human IPF and COVID-19 damaged lungs, loss of SOX2 correlates with the appearance of aberrant KRT5^−^/KRT17^+^ epithelial cells. Wnt signaling activation is also observed in these cells in IPF, suggesting cooperative action by these two pathways drive dysplastic remodeling in the fibrotic human lung. Our data define an essential role for Sox2-Wnt interactions in constraining airway epithelial cell fate which has broad impact on homeostasis and tissue regeneration and provide insights into the pathophysiology of lung fibrotic disease.

Restoring functional alveoli that can mediate gas exchange is a major challenge in lung regeneration following severe injury. Intrapulmonary airway cells can contribute to their own self-renewal, functional alveolar epithelial regeneration, or dysplastic remodeling depending on the severity of the injury. The dysplastic remodeling involves development of keratinized epithelial regions that do not facilitate functional gas exchange and can persist for up to one year after influenza infection in mouse models^9^. Understanding the molecular mechanisms of airway cell fate decisions during lung regeneration is critical to discover ways to achieve successful regeneration of a fully functional lung. Recent research indicates that the Notch signaling pathway, as well as the transcription factors Hif1α and ΔNp63, are essential for dysplastic alveolar repair, which is primarily achieved through the migration and proliferation of Trp63^+^ intrapulmonary basal-like progenitors^4,9,17^. Our current study highlights the role of Sox2 as a molecular brake that maintains airway cell identity and inhibits both functional and dysplastic regeneration of the alveoli. Limitation of our study is that we did not perform secretory cell lineage tracing and Trp63^+^ cell lineage tracing, however, many reports suggest that secretory cell-derived alveolar regeneration and Trp63^+^-derived dysplastic regeneration are two distinct responses to injury in the lung, and we believe that the use of Sox2^CreERT2^ encompasses both mechanisms^2–10^. There was no functional benefit of Sox2 pan-airway knockout for influenza infection; however, basaloid progenitor-specific Sox2 activation could theoretically be beneficial to promote functional regeneration in response to alveolar injury.

Sox2 exerts pleiotropic effects on cell identity, and its roles in the development and cancer across different organs have been reported^37,38^. During lung development, Sox2 is important for activation of Trp63 expression to regulate the emergence of basal cells^19,20,39,40^. Sox2 activation of Trp63 expression during development has also been reported in other organs, such as the stomach, suggesting that it is a conserved function of Sox2 during the development of multiple organs^41^. In contrast, our findings indicate that the deletion of Sox2 during adult homeostasis elevates Trp63 expression in basal-like progenitor cells, implying that Sox2 exerts antagonistic effects on Trp63 in adults. We think it is possible that loss of Sox2 disrupts the basal cell gene regulatory network and opens a new gene regulatory niche for Trp63, favoring Trp63 to amplify its own expression and effects. Interestingly, we found that Wnt activation alone at homeostasis enhances the proliferative activity of Trp63^+^ intrapulmonary basal-like progenitors, whereas Wnt activation during dysplastic regeneration inhibits migration of these cells into the alveolar space. The latter effect is overweighed by the simultaneous loss of Sox2 and activation of Trp63, a critical component of Trp63^+^ cell migration^9^. Wnt signaling functions in a context-dependent manner^42,43^, and further research is needed to understand the molecular basis of Wnt-Sox2-Trp63 interactions in Trp63^+^ cells during homeostasis and dysplastic regeneration.

The Wnt pathway effectors LEF/TCF and SOX transcription factors belong to the HMG domain protein and are structurally related^31^. SOX-Wnt interactions control the canonical Wnt signaling pathway, in part, through interactions with β-catenin which represses or activates canonical Wnt signaling^32^. Our study indicates that Sox2 regulates airway cell fate decisions, in part through its repression of Wnt signaling activity in secretory and BASC cell lineages. Previous report suggests that overexpression of Sox2 decreases the expression of Scgb1a1, suggesting that physiological levels of Sox2 expression are required for airway cell fate maintenance^44^. Recent scRNA-seq data shows that KRT5^−^/KRT17^+^ cells express high levels of SOX4^35^. In contrast to SOX2, SOX4 has been reported to enhance Wnt signaling activity, raising an interesting concept that differential SOX protein expression and activity could modulate Wnt signaling output in these airway epithelial lineages^31,45^. This could explain why we observed an enhanced Wnt transcriptional signature in dysplastic KRT5^−^/KRT17^+^ cells that are observed in IPF lungs. Our data show that IPF and post-COVID-19 are similar in that they both exhibit KRT5^−^/KRT17^+^/SOX2^−^ cells. However, IPF was unique in that these cells exhibit high expression of WNT signaling target genes. Nuclear β-catenin signal was prominent in KRT5^−^/KRT17^+^/SOX2^−^ cells surrounding fibroblastic foci, suggesting that this WNT-rich microenvironment at fibroblastic foci, together with increased SOX4 expression, play a pathological role in KRT17 + /KRT5- cells that lack SOX2 expression. Further investigation into what causes the dysplastic nature of KRT5^−^/KRT17^+^ cell state could lead to new approaches for treating lung disease.

## Methods

### Human subjects

The normal human samples used in this study were obtained from de-identified non-used lungs donated for organ transplantation according to an established protocol (PROPEL, approved by the Institutional Review Board of the University of Pennsylvania) with informed consent in accordance with institutional and NIH procedures. Consents were obtained from next of kin or health care proxy. Diseased tissue from patients with severe IPF or post-COVID-19 fibrosis was obtained from participants enrolled in the Prospective Registry of Outcomes in Patients Electing Lung Transplantation (PROPEL) (Penn cohort) at the University of Pennsylvania^46^. The University of Pennsylvania institutional review board approved this study, and all patient information was removed prior to use. This use does not meet the current NIH definition of human subject research, but all institutional procedures required for human subject research were followed throughout the reported experiments.

### Animals

All mouse experiments were performed in accordance with protocols approved under the guidance of the Institutional Animal Care and Use Committee of the University of Pennsylvania. *Sox2*^*CreERT2*^ (Stock No. 017593), *Sox2*^*Flox*^ (Stock No. 013093), and *Rosa26R*^*EYFP*^ (Stock No: 007903) mice have been previously described and were obtained from Jackson Laboratories (MA, USA)^47,48^. *Ctnnb1*^*flox(ex3)*^ mice were described previously^49^. Experiments were performed on 6-12 week old mice that were maintained on a mixed CD1 and C57BL/6 background. For all conditions, both male and female mice were used. Tamoxifen (Sigma-Aldrich) was dissolved in corn oil to prepare a stock at a concentration of 20 mg/mL. For gene knockout experiments, a dose of 200 mg/kg tamoxifen was administered by oral gavage for 3 consecutive days.

### Influenza injury

PR8-GP33 H1N1 influenza virus was provided by Dr. John Wherry. Two weeks after tamoxifen induction, mice were intranasally administered a dose of 1 LD_50_ (determined empirically in our laboratory) diluted in 50 μL sterile saline, except for *Sox2*^*CreERT2/Flox*^*; Ctnnb1*^*flox(ex3)/wt*^ and *Sox2*^*CreERT2*^*; Ctnnb1*^*flox(ex3)/wt*^ mice, which received a dose of 0.5 LD_50_. Peripheral oxygen saturation was measured using a MouseOx Plus rat & mouse pulse oximeter and a MouseOx small collar sensor (Starr Life Sciences Corp.) as previously explained^50^.

### Histology

Mice were euthanized by inhalation of CO_2_, and the lungs were perfused with PBS through the right ventricle. The lungs were then inflated with 2% PFA at a constant pressure of 25 cm H_2_O and were fixed overnight at 4 °C. Tissues were then dehydrated, embedded in paraffin, and sectioned. For immunohistochemistry, the following antibodies were used: GFP (chicken, Aves, GFP-1020, 1:200), Nkx2-1 (rabbit, Abcam, ab76013, 1:100), Sox2 (goat, R&D, AF2018, 1:50), Sox9 (rabbit, Abcam, ab185966, 1:100), Scgb1a1 (mouse, Santa Cruz, sc-365992, 1:100), SCGB1A1 (rat, R&D, MAB4218, 1:40), Scgb3a2 (mouse, Novus, H00117156-M01, 1:100), β-Tubulin IV (mouse, Biogenex, MU178-UC, 1:20), p63 (rabbit, Santa Cruz, sc-8343, 1:100), β-catenin (mouse, BD Biosciences, 610154, 1:100), Sftpc (rabbit, Millipore, AB3786, 1:200), SFTPC (rabbit, Abcam, ab90716, 1:100), Lamp3 (rat, Novus, 1010E1.01, 1:100), Hopx (mouse, Santa Cruz, sc-398703, 1:100), Krt5 (rabbit, Abcam, ab52635, 1:500), Krt5 (chicken, BioLegend, 905901, 1:100), Krt8 (rat, DSHB, TROMA-1, 1:100), and Krt17 (mouse, Santa Cruz, sc-393002, 1:200).

### Flow cytometry and cell sorting

A CytoFLEX LX cell analyzer (Beckman Coulter) and a MoFlo Astrios cell sorter (Beckman Coulter) were used for flow cytometry and cell sorting experiments. Using collagenase I, dispase, and DNase, lung tissue was harvested and digested into single cell suspensions, as previously explained^51–53^. ACK buffer was used to remove red blood cells followed by antibody staining. The following antibodies were used for flow cytometry and cell sorting: CD31-APC (eBioscience, 390, 1:200), CD45-APC (eBioscience, 30-F11, 1:200), and EpCAM-PE-Cy7 (eBioscience, G8.8, 1:200). Dead cells were excluded using DAPI.

### Organoid assay

Lineage-traced airway cells were sorted by gating on DAPI^–^ CD31/45-APC^–^ EpCAM-PECy7^+^ EYFP^+^ populations. For each well, 3 ×10^4^ airway cells were combined with 5 ×10^4^ primary lung fibroblasts and mixed at a 1:1 ratio with growth factor-reduced Matrigel (Corning) and plated (90 μL/well) on a 24-well cell culture insert (Thermo Fisher). After 20 min incubation at 37 °C to solidify Matrigel, 500 μL MTEC medium was added as previously described^54^. The MTEC medium was prepared by adding 5% FBS, cholera toxin (0.1 μg/mL, Sigma), EGF (25 ng/mL, Peprotech), and components of SAGM supplements (bovine pituitary extract, insulin, transferrin, gentamycin, and retinoic acid; Lonza) to DMEM/F12 base medium. Y-27632 Rho kinase inhibitor (10 μM, Sigma) was added to the medium for the first 48 h of culture, and the medium was changed every 48 h. A tankyrase inhibitor XAV939 (10 μM, Sigma) was added to the medium starting on day 2 of culture. Organoids were fixed in 2% PFA for 30 min at 4 °C, dehydrated, embedded in paraffin, and sectioned.

### Image analysis

Fluorescence images were acquired by a Leica Stellaris 5 or Leica TCS SP8 confocal microscope and processed with ImageJ. For each mouse, at least five z-stack images were collected with a 40x lens for cell counting. EYFP^+^ lineage-traced cells were used for cell differentiation analysis. For organoid quantification, organoids containing Krt5^+^ cells were considered basal organoids, and organoids containing Sftpc^+^ cells were considered alveolar organoids. Intrapulmonary basaloid progenitor quantification was performed in intrapulmonary airways within 1-2 bifurcations of the right or left intrapulmonary bronchus. Intrapulmonary airways were defined as airways in which we observed alveolar structure surrounding the airway. Krt5^+^ region was quantified by imaging the entire lobe using EVOS FL Auto2 Imaging System. The EVOS was also used to image organoids over the course of culture.

### Single cell RNA sequencing (scRNA-seq)

DAPI^–^ CD31/45-APC^–^ EpCAM-PECy7^+^ EYFP^+^ lineage-traced airway cells from control (one female mouse at homeostasis; one male and one female mouse after influenza infection) and Sox2^cKO^ (one female mouse at homeostasis; two male and two female mice after influenza infection) were sorted and were loaded onto the 10x Chromium (10X Genomics) aiming for 10,000 cells, and libraries were prepared according to the manufacturer’s protocol using Chromium Single Cell 3’ v3 chemistry. Illumina Novaseq 6000 instrument was used for sequencing. The raw reads were aligned using STARsolo (v2.7.9a)^55^. The number of cells used for analysis was 9270 cells for homeostasis control, 5923 cells for homeostasis Sox2^cKO^, 2940 cells for control after influenza infection, 4503 cells for Sox2^cKO^ after influenza infection. The data was further processed and analyzed using the Seurat (V4.0.6; https://satijalab.org/seurat/) package. Cells with less than 1000 UMIs were removed. Also, cells with potential stress signals were removed if the percent mitochondrial reads were greater than 5%. Samples were merged, and Feature (gene) data was scaled in order to remove unwanted sources of variation using the Seurat SCTransform function based on percent mitochondrial reads, and the number of genes and reads. Non-linear dimension reduction was performed using uniform manifold projection (UMAP) and graph-based clustering was performed using the Louvain. Trajectory inference was performed using the R package Slingshot^56^. AddModuleScore function was used to evaluate Wnt activation scores. Wnt target genes were extracted from the Wnt homepage (https://web.stanford.edu/group/nusselab/cgi-bin/wnt/), and non-expressed genes were excluded from the analysis.

### Statistics

Results are presented as means with standard deviations. Two groups were compared using a two-tailed t-test. Multiple groups were compared using a one-way ANOVA. The difference was considered significant when *p* < 0.05. Graphpad Prism 9 was used for statistical analysis.

### Reporting summary

Further information on research design is available in the Nature Research Reporting Summary linked to this article.

### Supplementary information

Supplemental Information

Supplementary Data 1

Supplementary Data 2

Reporting summary

## Data Availability

scRNA-seq data generated during this study have been deposited in the GEO database (GSE227905). scRNA-seq data for human samples^35^ and p63 ChIP-seq data^9^ are publicly available (GSE135893 for scRNA-seq and GSE216161 for ChIP-seq). All other data are available on request from the corresponding author.

## Code availability

Public software and custom code described in the Methods section were used to process the data in this paper.

## References

[1] Hogan BL (2014). Repair and regeneration of the respiratory system: complexity, plasticity, and mechanisms of lung stem cell function. Cell Stem Cell.

[2] Ray S (2016). Rare SOX2(+) Airway Progenitor Cells Generate KRT5(+) Cells that Repopulate Damaged Alveolar Parenchyma following Influenza Virus Infection. Stem Cell Rep..

[3] Zuo W (2015). p63(+)Krt5(+) distal airway stem cells are essential for lung regeneration. Nature.

[4] Vaughan AE (2015). Lineage-negative progenitors mobilize to regenerate lung epithelium after major injury. Nature.

[5] Kathiriya JJ, Brumwell AN, Jackson JR, Tang X, Chapman HA (2020). Distinct Airway Epithelial Stem Cells Hide among Club Cells but Mobilize to Promote Alveolar Regeneration. Cell Stem Cell.

[6] Liu Q (2019). Lung regeneration by multipotent stem cells residing at the bronchioalveolar-duct junction. Nat. Genet..

[7] Salwig I (2019). Bronchioalveolar stem cells are a main source for regeneration of distal lung epithelia in vivo. EMBO J..

[8] Kanegai CM (2016). Persistent Pathology in Influenza-Infected Mouse Lungs. Am. J. Respir. Cell Mol. Biol..

[9] Weiner AI (2022). DeltaNp63 drives dysplastic alveolar remodeling and restricts epithelial plasticity upon severe lung injury. Cell Rep..

[10] Yang Y (2018). Spatial-Temporal Lineage Restrictions of Embryonic p63(+) Progenitors Establish Distinct Stem Cell Pools in Adult Airways. Dev. Cell.

[11] Kim CF (2005). Identification of bronchioalveolar stem cells in normal lung and lung cancer. Cell.

[12] Weibel ER, Gomez DM (1962). Architecture of the human lung. Use of quantitative methods establishes fundamental relations between size and number of lung structures. Science.

[13] Weibel ER, Sapoval B, Filoche M (2005). Design of peripheral airways for efficient gas exchange. Respir. Physiol. Neurobiol..

[14] Basil MC (2022). Human distal airways contain a multipotent secretory cell that can regenerate alveoli. Nature.

[15] Kadur Lakshminarasimha Murthy P (2022). Human distal lung maps and lineage hierarchies reveal a bipotent progenitor. Nature.

[16] Rustam S (2023). A Unique Cellular Organization of Human Distal Airways and Its Disarray in Chronic Obstructive Pulmonary Disease. Am. J. Respir. Crit. Care Med..

[17] Xi Y (2017). Local lung hypoxia determines epithelial fate decisions during alveolar regeneration. Nat. Cell Biol..

[18] Danopoulos S (2018). Human lung branching morphogenesis is orchestrated by the spatiotemporal distribution of ACTA2, SOX2, and SOX9. Am. J. Physiol. Lung Cell Mol. Physiol..

[19] Gontan C (2008). Sox2 is important for two crucial processes in lung development: branching morphogenesis and epithelial cell differentiation. Dev. Biol..

[20] Que J, Luo X, Schwartz RJ, Hogan BL (2009). Multiple roles for Sox2 in the developing and adult mouse trachea. Development.

[21] Que J (2007). Multiple dose-dependent roles for Sox2 in the patterning and differentiation of anterior foregut endoderm. Development.

[22] Tompkins DH (2009). Sox2 is required for maintenance and differentiation of bronchiolar Clara, ciliated, and goblet cells. PLoS One.

[23] Frank DB (2016). Emergence of a Wave of Wnt Signaling that Regulates Lung Alveologenesis by Controlling Epithelial Self-Renewal and Differentiation. Cell Rep..

[24] Zacharias WJ (2018). Regeneration of the lung alveolus by an evolutionarily conserved epithelial progenitor. Nature.

[25] Chang DR (2013). Lung epithelial branching program antagonizes alveolar differentiation. Proc. Natl Acad. Sci. USA.

[26] Eenjes E (2021). SOX21 modulates SOX2-initiated differentiation of epithelial cells in the extrapulmonary airways. Elife.

[27] Choi J (2020). Inflammatory Signals Induce AT2 Cell-Derived Damage-Associated Transient Progenitors that Mediate Alveolar Regeneration. Cell Stem Cell.

[28] Kobayashi Y (2020). Persistence of a regeneration-associated, transitional alveolar epithelial cell state in pulmonary fibrosis. Nat. Cell Biol..

[29] Strunz M (2020). Alveolar regeneration through a Krt8+ transitional stem cell state that persists in human lung fibrosis. Nat. Commun..

[30] Goss AM (2009). Wnt2/2b and beta-catenin signaling are necessary and sufficient to specify lung progenitors in the foregut. Dev. Cell.

[31] Kormish JD, Sinner D, Zorn AM (2010). Interactions between SOX factors and Wnt/beta-catenin signaling in development and disease. Dev. Dyn..

[32] Mansukhani A, Ambrosetti D, Holmes G, Cornivelli L, Basilico C (2005). Sox2 induction by FGF and FGFR2 activating mutations inhibits Wnt signaling and osteoblast differentiation. J. Cell Biol..

[33] Barkauskas CE (2013). Type 2 alveolar cells are stem cells in adult lung. J. Clin. Invest..

[34] Rock JR (2009). Basal cells as stem cells of the mouse trachea and human airway epithelium. Proc. Natl Acad. Sci. USA.

[35] Habermann AC (2020). Single-cell RNA sequencing reveals profibrotic roles of distinct epithelial and mesenchymal lineages in pulmonary fibrosis. Sci. Adv..

[36] Adams TS (2020). Single-cell RNA-seq reveals ectopic and aberrant lung-resident cell populations in idiopathic pulmonary fibrosis. Sci. Adv..

[37] Novak D (2020). SOX2 in development and cancer biology. Semin. Cancer Biol..

[38] Wuebben EL, Rizzino A (2017). The dark side of SOX2: cancer - a comprehensive overview. Oncotarget.

[39] Hashimoto S (2012). beta-Catenin-SOX2 signaling regulates the fate of developing airway epithelium. J. Cell Sci..

[40] Ochieng JK (2014). Sox2 regulates the emergence of lung basal cells by directly activating the transcription of Trp63. Am. J. Respir. Cell Mol. Biol..

[41] Francis R (2019). Gastrointestinal transcription factors drive lineage-specific developmental programs in organ specification and cancer. Sci. Adv..

[42] Nusse R, Clevers H (2017). Wnt/beta-Catenin Signaling, Disease, and Emerging Therapeutic Modalities. Cell.

[43] Rim EY, Clevers H, Nusse R (2022). The Wnt Pathway: From Signaling Mechanisms to Synthetic Modulators. Annu. Rev. Biochem..

[44] Tompkins DH (2011). Sox2 activates cell proliferation and differentiation in the respiratory epithelium. Am. J. Respir. Cell Mol. Biol..

[45] Sinner D (2007). Sox17 and Sox4 differentially regulate beta-catenin/T-cell factor activity and proliferation of colon carcinoma cells. Mol. Cell Biol..

[46] Diamond JM (2013). Clinical risk factors for primary graft dysfunction after lung transplantation. Am. J. Respir. Crit. Care Med..

[47] Arnold K (2011). Sox2(+) adult stem and progenitor cells are important for tissue regeneration and survival of mice. Cell Stem Cell.

[48] Shaham O (2009). Pax6 is essential for lens fiber cell differentiation. Development.

[49] Harada N (1999). Intestinal polyposis in mice with a dominant stable mutation of the beta-catenin gene. EMBO J..

[50] Zhao G (2020). Regeneration of the pulmonary vascular endothelium after viral pneumonia requires COUP-TF2. Sci. Adv..

[51] Penkala IJ (2021). Age-dependent alveolar epithelial plasticity orchestrates lung homeostasis and regeneration. Cell Stem Cell.

[52] Shiraishi K (2023). Biophysical forces mediated by respiration maintain lung alveolar epithelial cell fate. Cell.

[53] Zepp JA (2017). Distinct Mesenchymal Lineages and Niches Promote Epithelial Self-Renewal and Myofibrogenesis in the Lung. Cell.

[54] Liberti DC (2021). Alveolar epithelial cell fate is maintained in a spatially restricted manner to promote lung regeneration after acute injury. Cell Rep..

[55] Dobin A (2013). STAR: ultrafast universal RNA-seq aligner. Bioinformatics.

[56] Street K (2018). Slingshot: cell lineage and pseudotime inference for single-cell transcriptomics. BMC Genomics.

