# Supplementary material for: Airway epithelial cell identity and plasticity are constrained by Sox2 during lung homeostasis, tissue regeneration, and in human disease
Source: NPJ Regen Med. 2024 Jan 5;9:2. doi: 10.1038/s41536-023-00344-w (PMC10770358; doi:10.1038/s41536-023-00344-w)
Supplement: Supplementary file 1 — Supplemental Information [file 41536_2023_344_MOESM1_ESM.pdf]

Supplementary Figure 1

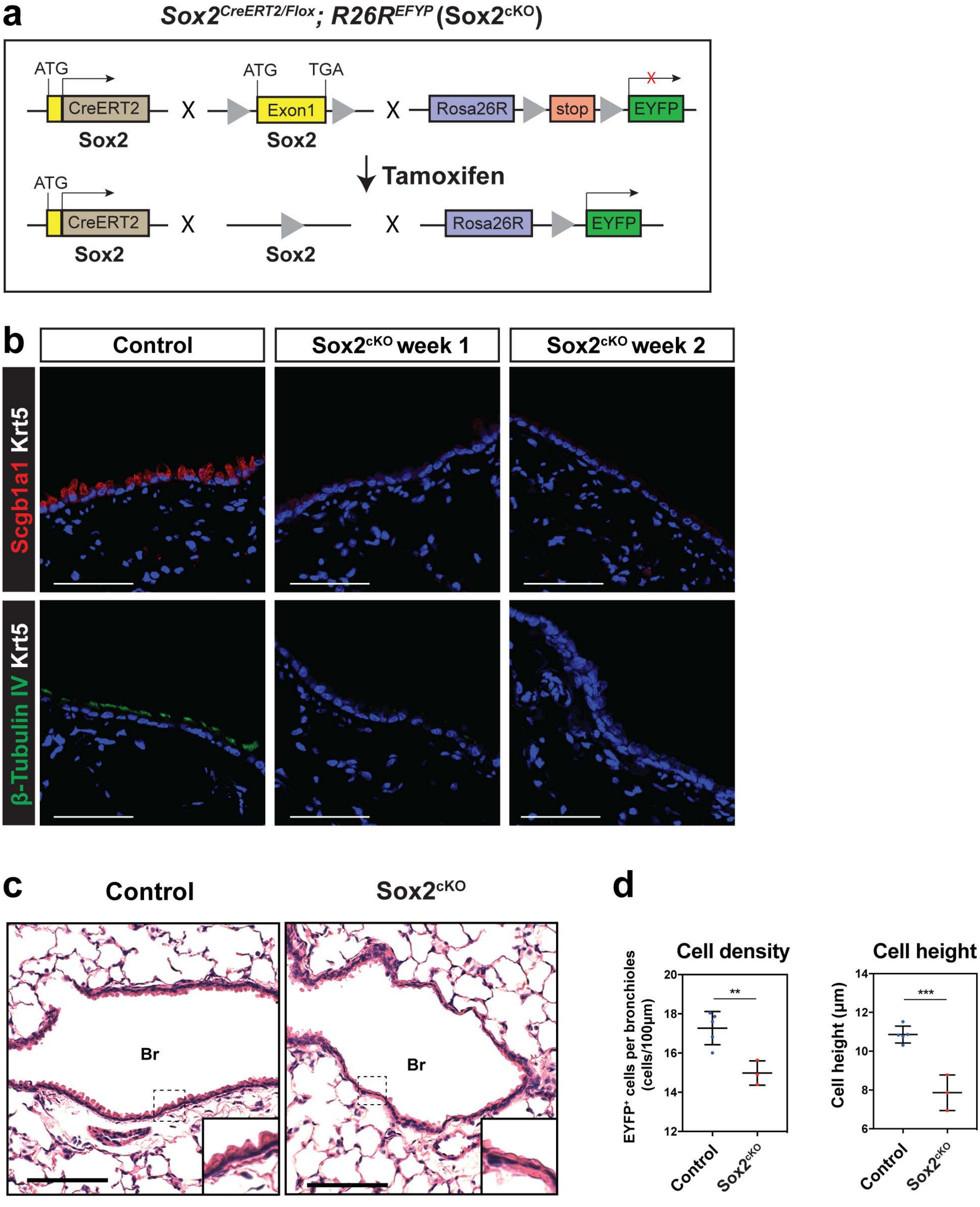

Supplementary Figure 1. Sox2 conditional knockout strategy

a. Diagram illustrates strategy for deleting Sox2 in airway cells using *Sox2$^{CreERT2/Flox}$; R26R^{EYFP}* mice.

b. Control and Sox2^{cKO} mice were given tamoxifen and analyzed 7 or 14 days later. Secretory cell marker Scgb1a1 and ciliated cell marker β-Tubulin IV are downregulated in Sox2^{cKO} mice.

c. Representative HE pictures from control and Sox2^{cKO} mice.

d. Quantification for the cell density and cell height. N = 5 control and n = 3 Sox2^{cKO} mice. *** $P < 0.001$ and ** $P < 0.01$ by two-tailed t-test. Each dot represents an individual mouse, and error bars indicate mean with s.d.

Scale bars: c, 100 μm; b, 50 μm.

Supplementary Figure 2

a

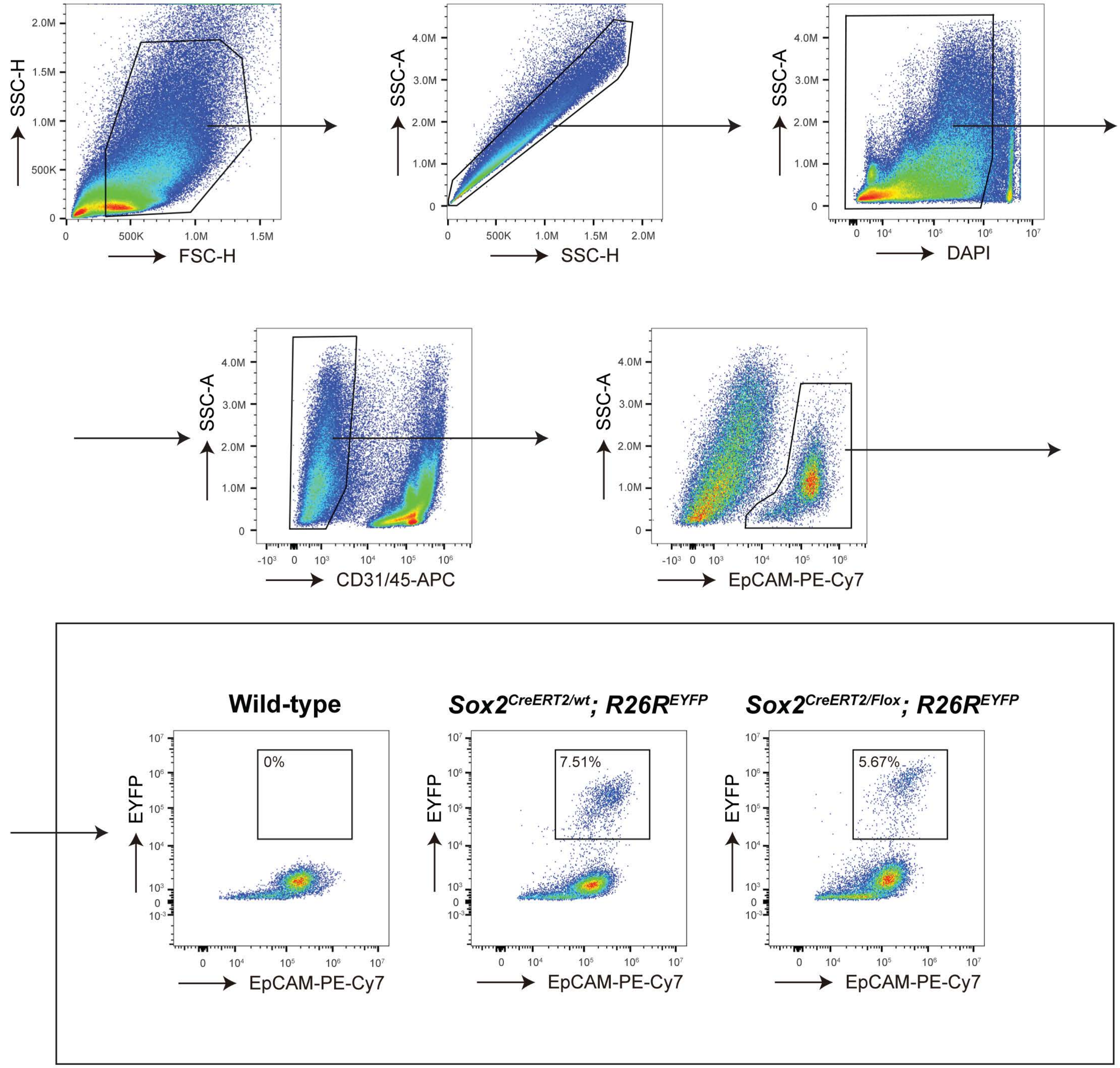

b

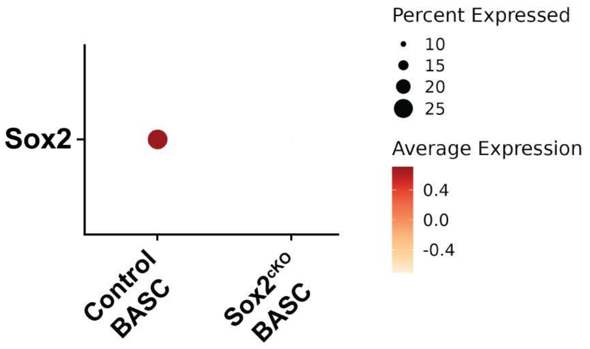

Supplementary Figure 2. FACS gating strategy and Sox2 expression in BASCs

a. A representative FACS gating strategy that was used in this study for the isolation of $DAPI^{–}$ $CD31^{–}$ $CD45^{–}$ $EpCAM^{+}$ $EYFP^{+}$ airway cells from $Sox2^{CreERT2/wt}$; $R26R^{EYFP}$ (control) and $Sox2^{CreERT2/Flox}$; $R26R^{EYFP}$ ($Sox2^{cKO}$) mice.

b. Dotplot showing Sox2 expression in BASCs.

Supplementary Figure 3

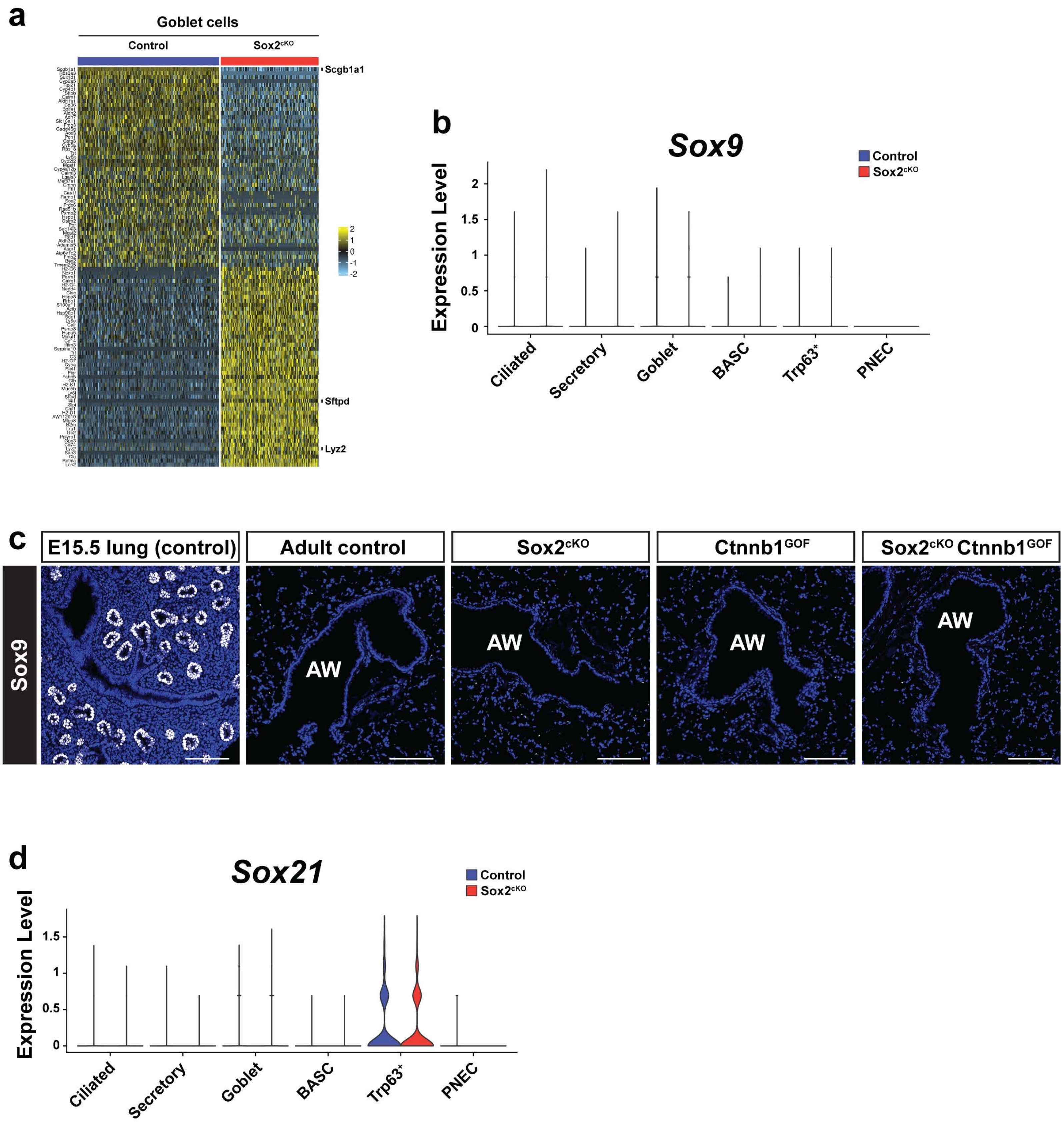

Supplementary Figure 3. Additional scRNA-seq analysis

a. Heatmap showing the top 50 differentially expressed genes for goblet cells.

b. Violin plot showing Sox9 expression in control and $Sox2^{cKO}$ mice.

c. Control, $Sox2^{cKO}$, $Ctnnb1^{GOF}$ (related to Figure 5), and $Sox2^{cKO}$/$Ctnnb1^{GOF}$ (related to Figure 5) lungs were negative for Sox9. Scale bars: 100 μm.

d. Violin plot showing Sox21 expression in control and $Sox2^{cKO}$ mice.

Supplementary Figure 4

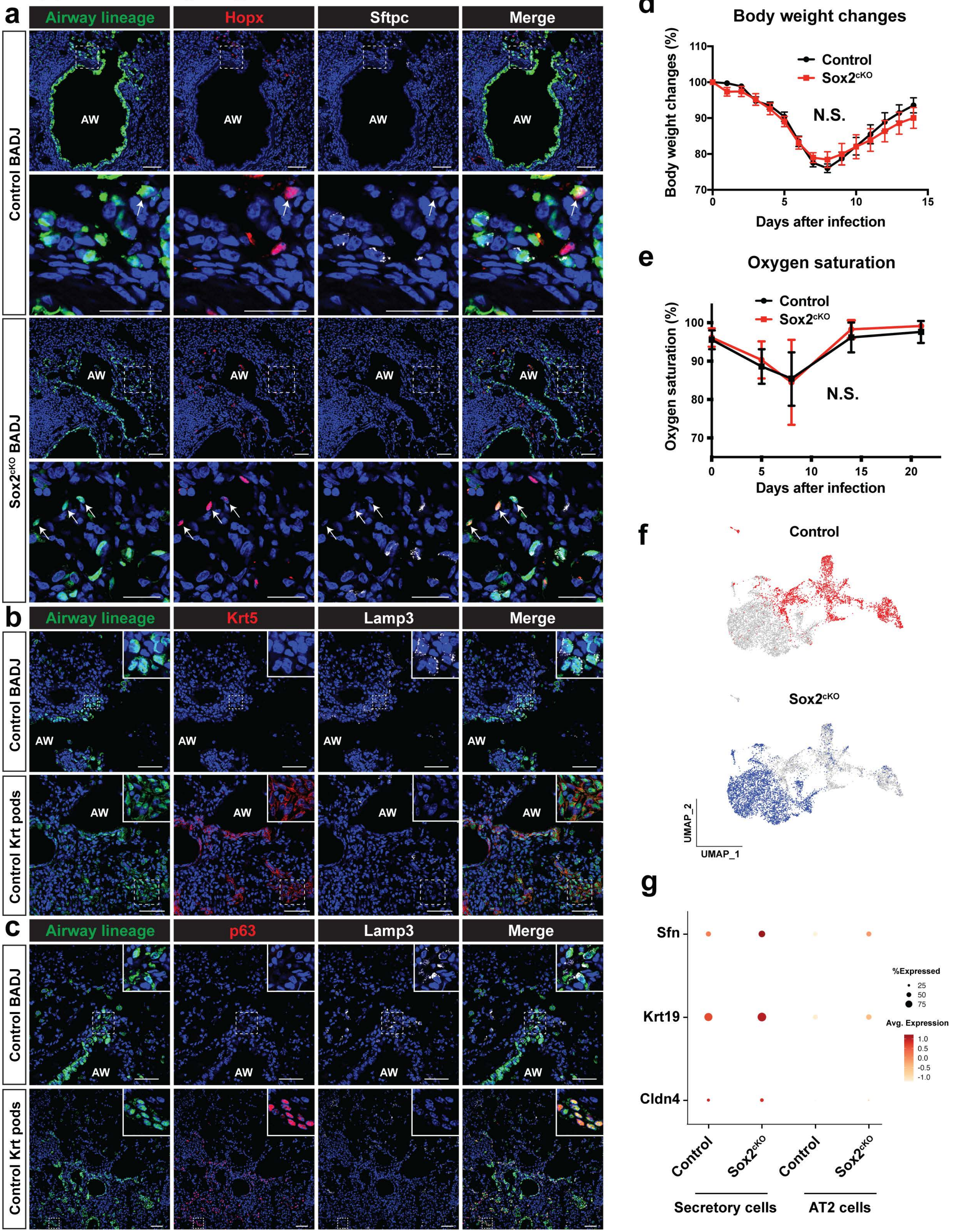

Supplementary Figure 4. Additional analysis on influenza infection experiment

a. Low magnification and high magnification images from influenza infection showing airway-derived Hopx$^+$ AT1 cells. Arrows indicate AT1 cells.

b-c. Airway-derived AT2 cells do not express Krt5 (b) or p63 (c). Krt5$^+$ (b) or p63$^+$ (c) dysplastic epithelium does not express AT2 cell marker Lamp3.

d. There were no changes in body weight trends after influenza infection. N = 13 control and n = 11 Sox2^{cKO} mice from 6 experiments.

e. Measurement of oxygen saturation in n = 8 control and n = 12 Sox2^{cKO} mice.

f. UMAP showing lineage traced populations of EYFP$^+$ cells from control (red) and Sox2^{cKO} (blue) mice 28 days after influenza infection.

g. Dotplot showing expression of Sfn, Krt19, and Cldn4 in secretory and AT2 cells after influenza infection.

Scale bars: a (low magnification), b, and c, 50 μm; a (high magnification), 25 μm.

N.S., not significant, $P > 0.05$ by two-tailed t-test. Each dot represents an individual mouse, and error bars indicate mean with s.d.

Supplementary Figure 5

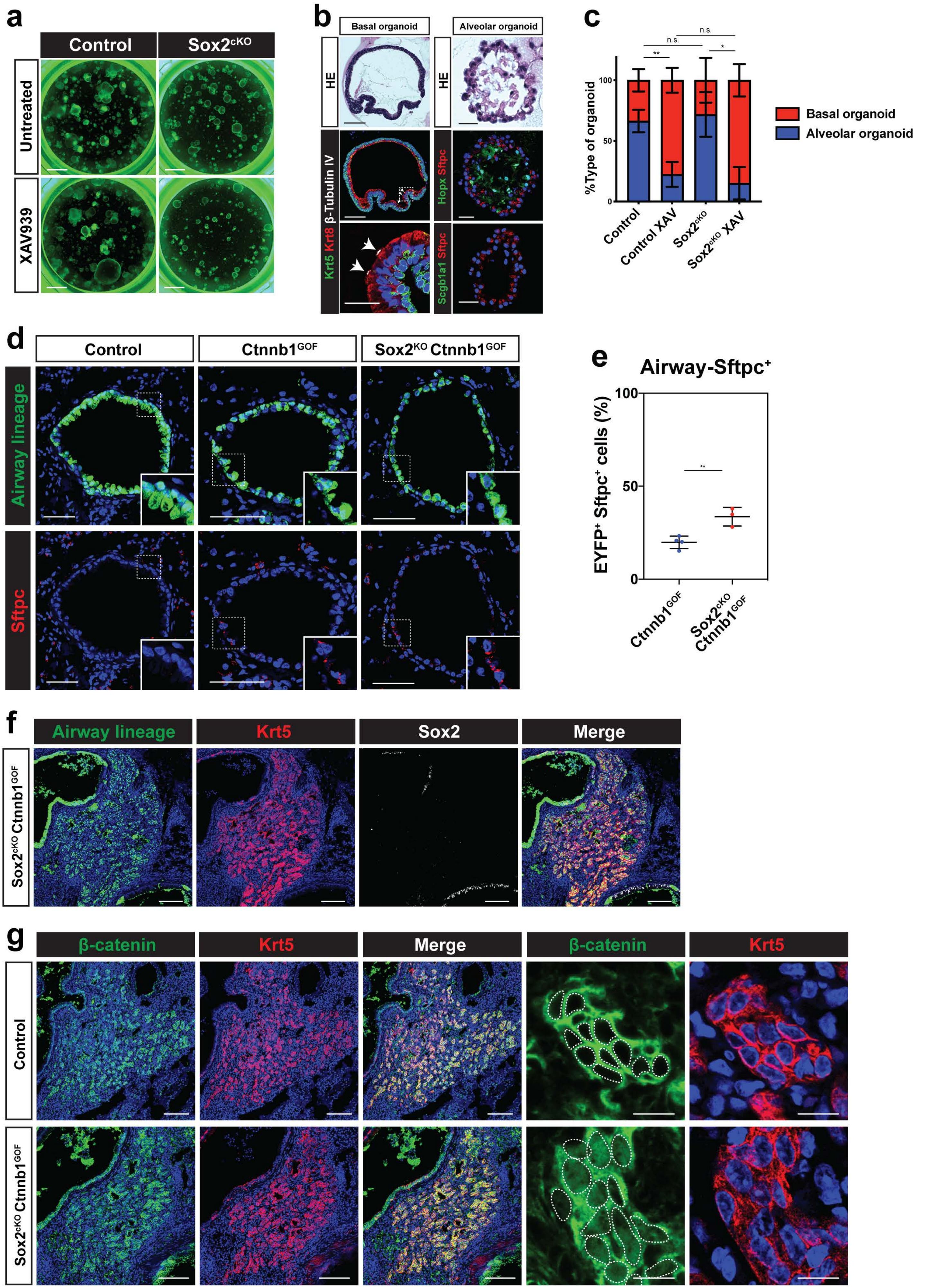

Supplementary Figure 5. Organoid experiment and additional analysis on Ctnnb1^{GOF} and Sox2^{cKO}/Ctnnb1^{GOF} mice

a. Airway cells from *Sox2$^{CreERT2/wtx}$; R26R^{EYFP}* and *Sox2$^{CreERT2/Flox}$; R26R^{EYFP}* mice were sorted, and organoid assays were performed. XAV939 treatment inhibited alveolar organoid formation from control and Sox2^{cKO}-derived airway cells (bottom).

b. Representative basal and alveolar organoids.

c. Quantification for the composition of Krt5^{+} basal and Sftpc^{+} alveolar organoids within the well. Organoids on the same slide were concurrently stained for Krt5 and Sftpc. Control (n = 4 wells from n = 3 experiments), Sox2^{cKO} (n = 3 wells and experiments), control airway cells treated with XAV939 (n = 3 wells and experiments), Sox2^{cKO} airway cells treated with XAV939 (n = 3 wells and experiments).

d-e. Ctnnb1^{GOF} and Sox2^{cKO}/Ctnnb1^{GOF} airway cells express Sftpc during homeostasis (d). Quantification of Sftpc^{+} cells during homeostasis from n = 4 Ctnnb1^{GOF} and n = 3 Sox2^{cKO}/Ctnnb1^{GOF} mice (e).

f. Tiled image showing that Sox2 is not expressed in Krt5^{+} dysplastic epithelium from Sox2^{cKO}/Ctnnb1^{GOF} mice.

g. Krt5^{+} dysplastic epithelium from Sox2^{cKO}/Ctnnb1^{GOF} mice shows nuclear β-catenin signal. Left three images are tiled images.

Scale bars: a, 1 mm; b (left), f, g (left three), 100 μm; d, 50 μm; b (right), 25 μm; g (right two), 10 μm.

** $P < 0.01$, and * $P < 0.05$ by two-tailed t-test. N.S., not significant, $P > 0.05$ by two-tailed t-test. Each dot represents an individual mouse or well, and error bars indicate mean with s.d.

Supplementary Figure 6

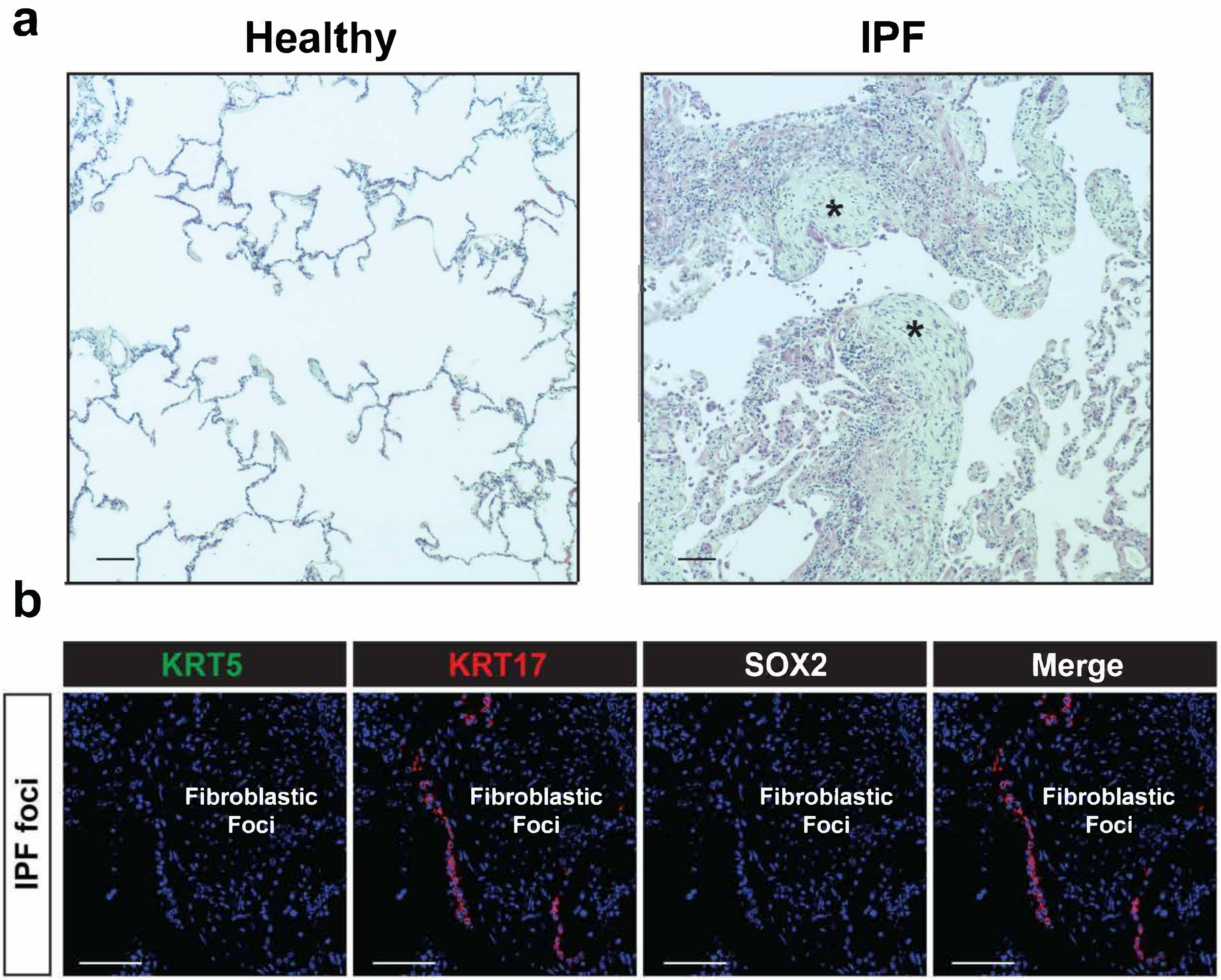

Supplementary Figure 6. Additional histological evaluation of IPF

a. H&E staining of healthy and IPF lung tissue. IPF tissue shows fibroblastic foci and thickened alveoli.

Asterisks denote fibroblastic foci.

b. $KRT5^{-}$/$KRT17^{+}$ dysplastic cells are $TP63^{+}$ at the fibroblastic foci.

Scale bars: a, b, 100 μm.

Supplementary Figure 7

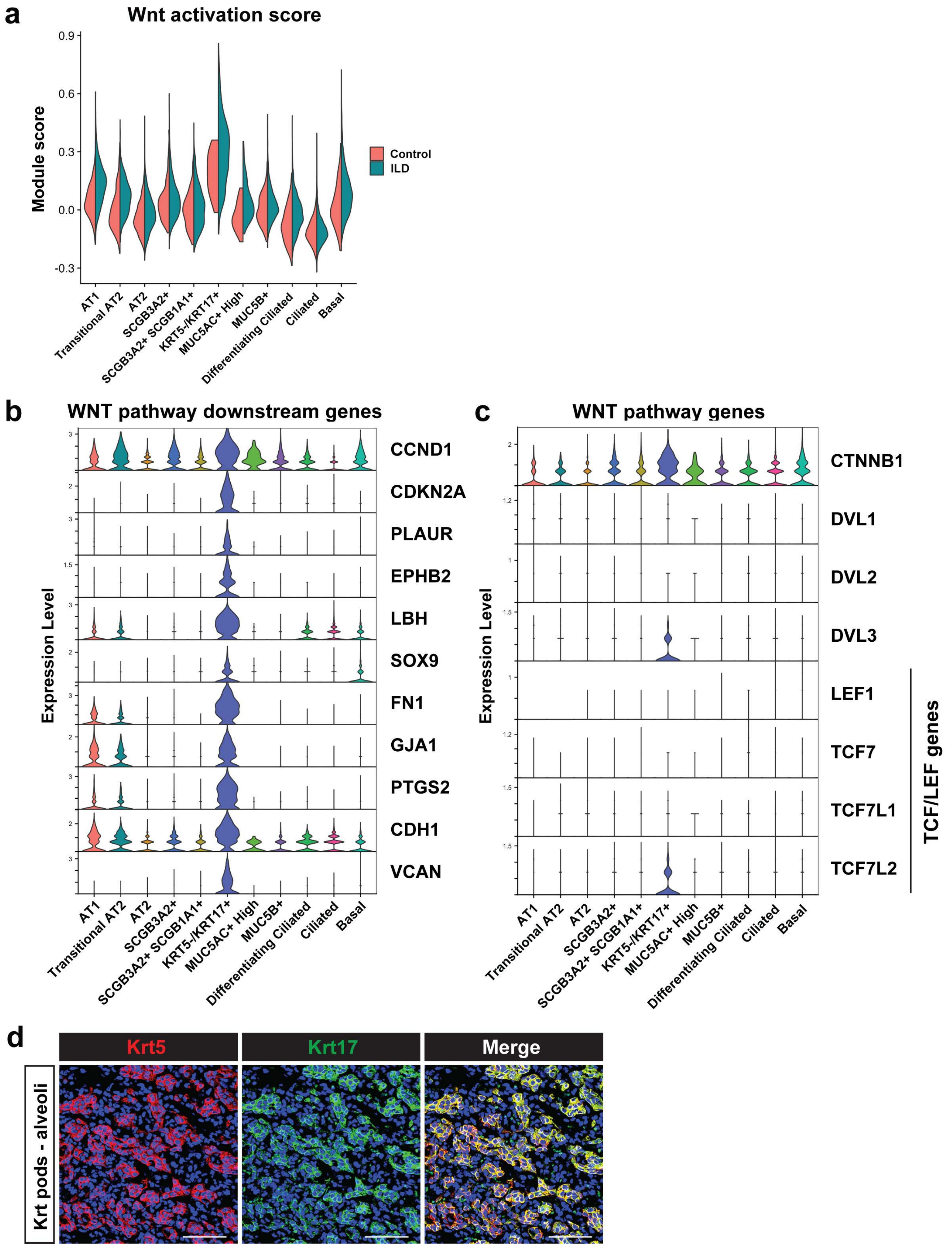

Supplementary Figure 7. WNT pathway and downstream gene expression in KRT17$^+$ dysplastic epithelial cells

a. Violin plot showing Wnt activation scores comparing epithelial cells from healthy controls and patients with pulmonary fibrosis.

b. Violin plots showing higher expression of WNT pathway downstream genes in KRT5$^-$/KRT17$^+$ dysplastic epithelial cells.

c. Violin plots showing higher expression of CTNNB1, DVL3, and TCF7L2 in KRT5$^-$/KRT17$^+$ dysplastic epithelial cells.

d. Mouse Krt5$^+$ epithelium also expresses Krt17. Scale bars: 50 μm.

Supplementary Table 1. Module genes

AT2 transciptional signature genes used for Figure 2
Sftpc
Lamp3
Slc34a2
Sftpb
Sftpa1
Cxcl15
Npc2
Dram1
Sfta2
Bex4
Ppp1r14c
Abca3
Sftpd

Wnt target genes used for Figure 3
Myc
Ccnd1
Jun
Tcf4
Cd44
Ephb2
Bmp4
Vegfa
Met
Mycbp
Id2
Jag1
Msl1
Tiam1
Nos2
Lbh
Ccn1
Pttg1
Dll1
Fn1
Gja1
Gjb6
Rarg
Enpp2
Tbx1
Ptgs2
Irx3
Emp1
Emp2
Btrc
Egfr
Cdh1
Klf5
Ret
Krt5
Krt14
Fgfr2

WNT target genes used for Figure 7
MYC
CCND1
JUN
PLAUR
MMP7
TCF4
CD44
EPHB2
BMP4
CLDN1
BIRC5
VEGFA
MET
EDN1
MYCBP
ID2
JAG1
MSL1
LBH
SOX9
RUNX2
CYR61
SOX2
PTTG1
FN1
GJA1
RHOU
TNFSF9
PTGS2
IRX3
EMP1
EMP2
EGFR
CDH1
CDKN2A
KLF5
CXCL8
VCAN
TNFRSF19
KRT5

Supplementary Table 2. Patient characteristics

ID	AGE	Cause of Death / Disease at Transplant	GENDER	RACE
Donor_1	53	N/A	Female	White/Caucasian
Donor_2	57	N/A	Male	Hispanic/Latino
Donor_3	76	N/A	Female	White/Caucasian
Donor_4	48	N/A	Male	White/Caucasian
IPF_1	65	IPF	Male	East Indian
IPF_2	69	IPF	Male	White/Caucasian
IPF_3	68	IPF	Male	White/Caucasian
IPF_4	58	IPF	Male	White/Caucasian
IPF_5	70	IPF	Male	White/Caucasian
COVID19_1	54	Post-COVID fibrosis	Male	White/Caucasian
COVID19_2	53	Post-COVID fibrosis	Male	White/Caucasian
COVID19_3	54	Post-COVID fibrosis	Male	White/Caucasian
