# Supplementary material for: Airway epithelial cell identity and plasticity are constrained by Sox2 during lung homeostasis, tissue regeneration, and in human disease
Source: NPJ Regen Med. 2024 Jan 5;9:2. doi: 10.1038/s41536-023-00344-w (PMC10770358; doi:10.1038/s41536-023-00344-w)
Supplement: Supplementary file 4 — Reporting summary [file 41536_2023_344_MOESM4_ESM.pdf]

Corresponding author(s): Edward E. Morrisey
Last updated by author(s): December 3, 2023

Reporting Summary

Nature Portfolio wishes to improve the reproducibility of the work that we publish. This form provides structure for consistency and transparency in reporting. For further information on Nature Portfolio policies, see our Editorial Policies and the Editorial Policy Checklist.

Statistics

For all statistical analyses, confirm that the following items are present in the figure legend, table legend, main text, or Methods section.

n/a	Confirmed	
☐	☒	The exact sample size (n) for each experimental group/condition, given as a discrete number and unit of measurement
☐	☒	A statement on whether measurements were taken from distinct samples or whether the same sample was measured repeatedly
☐	☒	The statistical test(s) used AND whether they are one- or two-sided<br>*Only common tests should be described solely by name; describe more complex techniques in the Methods section.*
☐	☒	A description of all covariates tested
☐	☒	A description of any assumptions or corrections, such as tests of normality and adjustment for multiple comparisons
☐	☒	A full description of the statistical parameters including central tendency (e.g. means) or other basic estimates (e.g. regression coefficient) AND variation (e.g. standard deviation) or associated estimates of uncertainty (e.g. confidence intervals)
☐	☒	For null hypothesis testing, the test statistic (e.g. F, t, r) with confidence intervals, effect sizes, degrees of freedom and P value noted<br>*Give P values as exact values whenever suitable.*
☒	☐	For Bayesian analysis, information on the choice of priors and Markov chain Monte Carlo settings
☒	☐	For hierarchical and complex designs, identification of the appropriate level for tests and full reporting of outcomes
☒	☐	Estimates of effect sizes (e.g. Cohen's d, Pearson's r), indicating how they were calculated

Our web collection on statistics for biologists contains articles on many of the points above.

Software and code

Policy information about availability of computer code

Data collection	Single cell RNA sequencing was done with a 10x genomics pipeline and analysis is described below. A CytoFLEX LX cell analyzer (Beckman Coulter) and a MoFlo Astrios cell sorter (Beckman Coulter) were used for flow cytometry and cell sorting experiments
Data analysis	Data was analyzed via Graphpad Prism 9, R, Image J, and FlowJo v10. R code used was described in detail and includes several previously published pipelines including STARsolo and Seurat V4. Our full code will be available upon request. Image analysis with Image J are described in the method section. FlowJo v10 was used for flow cytometry analysis.

For manuscripts utilizing custom algorithms or software that are central to the research but not yet described in published literature, software must be made available to editors and reviewers. We strongly encourage code deposition in a community repository (e.g. GitHub). See the Nature Portfolio guidelines for submitting code & software for further information.

Data

Policy information about availability of data

All manuscripts must include a data availability statement. This statement should provide the following information, where applicable:

- Accession codes, unique identifiers, or web links for publicly available datasets
- A description of any restrictions on data availability
- For clinical datasets or third party data, please ensure that the statement adheres to our policy

All data used to draw conclusions in the paper are present in the paper as described in the Data Availability section. All related data are available upon request from the corresponding author.

Research involving human participants, their data, or biological material

Policy information about studies with human participants or human data. See also policy information about sex, gender (identity/presentation), and sexual orientation and race, ethnicity and racism.

Reporting on sex and gender	Sex and gender of all patients are shown in Supplementary Table S4.
Reporting on race, ethnicity, or other socially relevant groupings	Race and ethnicity of all patients are shown in Supplementary Table S4.
Population characteristics	Detailed patient characteristics are shown in Supplementary Table S4. Normal lungs are de-identified non-used lungs for transplant. Diseased lungs came from University of Pennsylvania PROPEL cohort.
Recruitment	Normal lungs donated from organ transplant are recruited via Gift of Life organ donation program. Diseased lungs from organ transplant are recruited through the University of Pennsylvania PROPEL. Obtaining tissue from patients who chose to undergo transplantation may introduce confounding by socioeconomic status or race, but we do not believe this would affect the conclusions of this study.
Ethics oversight	The University of Pennsylvania institutional review board approved this study

Note that full information on the approval of the study protocol must also be provided in the manuscript.

Field-specific reporting

Please select the one below that is the best fit for your research. If you are not sure, read the appropriate sections before making your selection.

☒ Life sciences ☐ Behavioural & social sciences ☐ Ecological, evolutionary & environmental sciences

For a reference copy of the document with all sections, see nature.com/documents/nr-reporting-summary-flat.pdf

Life sciences study design

All studies must disclose on these points even when the disclosure is negative.

Sample size	For animal experiments, multiple animals were tested in 2-3 separate experiments in order to control for variation within and across cohorts. N=5 mice was chosen empirically as a baseline. Statistics were calculated across all biological and technical replicates.
Data exclusions	No data were excluded.
Replication	There were no difficulties reproducing any of the reported findings. This study includes multiple biological and technical replicates, with N and replication numbers reported in the legend of each figure.
Randomization	The experimental and control animals in all animal experiments were selected at random from littermates with the appropriate genotype. All animals used in these studies were maintained on a similar mixed C57Bl/6 x CD1 genetic background. We used both males and females for this study, and the results are representative of data obtained from animals of both sexes.
Blinding	Blinding to experimental condition was challenging because the genotype of all animals was known to the investigators.

Reporting for specific materials, systems and methods

We require information from authors about some types of materials, experimental systems and methods used in many studies. Here, indicate whether each material, system or method listed is relevant to your study. If you are not sure if a list item applies to your research, read the appropriate section before selecting a response.

Materials & experimental systems

n/a	Involved in the study	
☐	☒	Antibodies
☒	☐	Eukaryotic cell lines
☒	☐	Palaeontology and archaeology
☐	☒	Animals and other organisms
☒	☐	Clinical data
☒	☐	Dual use research of concern
☒	☐	Plants

Methods

n/a	Involved in the study	
☒	☐	ChIP-seq
☐	☒	Flow cytometry
☒	☐	MRI-based neuroimaging

Antibodies

Antibodies used	IHC: GFP (chicken, Aves, GFP-1020), Nkx2-1 (rabbit, Abcam, ab76013), Sox2 (goat, R&D, AF2018), Sox9 (rabbit, Abcam, ab185966), Scgb1a1 (mouse, Santa Cruz, sc-365992), SCGB1A1 (rat, R&D, MAB4218), Scgb3a2 (mouse, Novus, H00117156-M01), β-Tubulin IV (mouse, Biogenex, MU178-UC), p63 (rabbit, Santa Cruz, sc-8343), β-catenin (mouse, BD Biosciences, 610154), Sftpc (rabbit, Millipore, AB3786), SFTPC (rabbit, Abcam, ab90716), Lamp3 (rat, Novus, 1010E1.01), Hopx (mouse, Santa Cruz, sc-398703), Krt5 (rabbit, Abcam, ab52635), Krt5 (chicken, BioLegend, 905901), Krt8 (rat, DSHB, TROMA-1), and Krt17 (mouse, Santa Cruz, sc-393002).<br><br>Flow cytometry: EpCAM-PE-Cy7 (eBioscience, G8.8), CD31-APC (eBioscience, 390), and CD45-APC (eBioscience, 30-F11).
Validation	IHC<br>-All antibodies are widely and routinely used in the field and well validated. GFP (49 publications), Nkx2-1 (146 publications), Sox2 (63 publications), Sox9 (176 publications), Scgb1a1 (71 publications), SCGB1A1 (13 publications), Scgb3a2 (PMID: 35355013), β-Tubulin IV (8 publications), p63 (10 publications), β-catenin (6 publications), Sftpc (265 publications), SFTPC (70 publications), Lamp3 (at least 7 publications), Hopx (55 publications), Krt5 (rabbit, 139 publications; chicken, at least 5 publications), Krt8 (47 publications), and Krt17 (7 publications).<br><br>Flow cytometry<br>-EpCAM-PE-Cy7, CD31-APC, and CD45-APC are widely used flow cytometry antibodies across many fields. EpCAM-PE-Cy7 (at least 46 publications), CD31-APC (at least 26 publications), CD45-APC (at least 303 publications) according to the company websites. Gating plots are shown in the Extended data.

Animals and other research organisms

Policy information about studies involving animals; ARRIVE guidelines recommended for reporting animal research, and Sex and Gender in Research

Laboratory animals	Sox2CreERT2 (Stock No. 017593), Sox2Flox (Stock No. 013093), Ctnnb1flox(ex3), and Rosa26REYFP (Stock No: 007903) mice were used. Ctnnb1flox(ex3) was generously provided by Taketo lab (PMID: 10545105), and other strains were obtained from Jackson Laboratories (MA, USA). All experiments were performed on 6-12 week old mice that were maintained on a mixed C57BL/6 and CD1 background. Both male and female mice were used in all conditions.
Wild animals	The study did not include wild animals.
Reporting on sex	Both male and female mice were used in all conditions.
Field-collected samples	The study did not include field-collected samples.
Ethics oversight	All mouse experiments were performed under the protocols approved by the guidance of the University of Pennsylvania Institutional Animal Care and Use Committee.

Note that full information on the approval of the study protocol must also be provided in the manuscript.

Flow Cytometry

Plots

Confirm that:

☒ The axis labels state the marker and fluorochrome used (e.g. CD4-FITC).

☒ The axis scales are clearly visible. Include numbers along axes only for bottom left plot of group (a 'group' is an analysis of identical markers).

☒ All plots are contour plots with outliers or pseudocolor plots.

☒ A numerical value for number of cells or percentage (with statistics) is provided.

Methodology

Sample preparation	Samples were prepared by methods described in the methods section. In brief, lungs were digested into single cell suspensions using collagenase I, dispase, and DNase and stained with flow cytometry antibodies.
Instrument	Analysis was done on CytoFLEX LX (Beckman Coulter) and cell sorting was done on MoFlo Astrios (Beckman Coulter).
Software	FlowJo v10 was used for analysis.
Cell population abundance	Post-sort purity of EYFP positive cells was checked by widefield microscope for their EYFP positivity. Typically, EYFP negative cells were not observed or were negligible by microscopic observation.
Gating strategy	Gating strategy, including FSC/SSC gating, doublet exclusion gating, wildtype control, is described in the extended data.

☒ Tick this box to confirm that a figure exemplifying the gating strategy is provided in the Supplementary Information.
